# Chronic NH_4_Cl loading improves glucose tolerance without modifying insulin sensitivity in mice

**DOI:** 10.1038/s41598-026-38007-7

**Published:** 2026-02-03

**Authors:** Nawel Zaibi, Jessica Montaigne, Jennifer Baraka-Vidot, Judith Merrheim, Claire Devos, Emilie Caron, Florent Auger, Emmanuelle Durand, Bénédicte Toussaint, Souhila Amanzougarene, Mehdi Derhourhi, Philippe Froguel, Amélie Bonnefond, Régine Chambrey, Christophe Breton

**Affiliations:** 1 Inserm UMR1283, CNRS UMR8199, European Genomic Institute for Diabetes (EGID), Institut Pasteur de Lille, Lille University Hospital, Lille, France; 2 Université de Lille, Lille, France; 3 Université de Lille, Inserm, CHU Lille, U1172 LilNCog-Lille Neuroscience & Cognition, Lille, France; 4 Department of Metabolism, Imperial College London, London, UK

**Keywords:** Gene expression, Gluconeogenesis, Glucose homeostasis, Metabolic parameters, pH regulation, Renal function, Biochemistry, Diseases, Endocrinology, Physiology

## Abstract

Acute metabolic acidosis (MA), a feature mostly associated with chronic kidney disease, decreases glucose tolerance and insulin sensitivity. By contrast, the effects of chronic MA on glucose homeostasis remain elusive. Here, we evaluated glucose homeostasis and metabolic parameters in male mice exhibiting chronic MA via long-term NH_4_Cl administration. Unlike acute MA, chronic MA resulted in lower body weight, increased energy expenditure, lower basal glycemia, improved glucose tolerance without changes in insulin secretion or sensitivity. No overall glucose uptake changes were observed. However, hepatic and intestinal gluconeogenesis were decreased whereas renal endogenous glucose production was increased in mice with chronic MA. The elevated glucose urinary excretion was associated with lower expression of renal sodium/glucose co-transporters. Transcriptomic analysis revealed that chronic MA potentiates anion transport, glucose and lipid metabolism, mitochondrial and oxidative phosphorylation pathways in the kidney. Overall, chronic MA improves glucose tolerance without changes in insulin secretion or sensitivity, likely due to reduced hepatic gluconeogenesis, decreased renal glucose reabsorption and increased energy demands in the kidney.

**Supplementary Information:**

The online version contains supplementary material available at 10.1038/s41598-026-38007-7.

## Introduction

The global incidence of chronic kidney disease (CKD) which involves a gradual loss of kidney functions***,*** has reached a considerable number over the past years^1^, along with its complications. Acute metabolic acidosis (MA) is well known to be associated with CKD^2^, although few of these patients receive therapy to correct the pH unbalance^3^. Additionally, MA has been shown to lead to insulin resistance and/or impaired glucose homeostasis^4–8^, decreasing further the quality of life of patients.

Indeed, MA most commonly develops as CKD progresses due to a reduced acid excretion capacity and/or high daily exogenous acid load, resulting in a positive H^+^ balance^9^. As the number of viable nephrons decreases during disease progression^10^, single nephron acid excretion, especially via ammoniagenesis improves in some cases despite the reduced net renal acid excretion^11^. Single nephron acid excretion is enhanced, but not enough to compensate for the daily acid load, leading to a lost acid base balance^5^ and reduced bicarbonate concentration^12^. Serum bicarbonate concentration reduction has been associated with impaired fasting glucose and a higher risk of type 2 diabetes (T2D) in patients^13^. Ammonium chloride (NH_4_Cl) administration in healthy dogs demonstrated the impact of acute MA on decreased glucose uptake, causing chronic hyperglycemia linked to insulin resistance^14–16^. In healthy individuals, NH_4_Cl treatment led to insulin resistance after only 3 days of treatment^17,18^, which could partially be due to impaired insulin signaling via phosphoinositide 3-kinase (PI3K) in the muscle^19^, potentially associated with increased proteolysis, known to occur during MA^20^. Furthermore, acute MA led to decreased insulin receptors in adipocytes^21^, and decreased plasma adiponectin and leptin levels^22,23^. In line with these findings, the correction of MA by sodium bicarbonate (NaHCO_3_) supplementation in CKD patients not only decreased the progression of kidney failure, but reduced plasma insulin concentration and lowered the need for anti-diabetic drugs^7^, showing a clear relationship between blood pH and glucose homeostasis.

Over the past years, most functional studies have assessed the effects of acute MA^14,18–24^. By contrast, the effects of chronic MA on glucose tolerance and insulin resistance remain elusive. Questioning the effects of chronic MA in animal models may provide valuable information on underlying mechanisms resulting in glucose homeostasis deregulation and higher T2D risk in patients with long term acid–base balance disruption. Here, we evaluated metabolic parameters and glucose homeostasis in mice exhibiting chronic MA.

## Results

### Chronic NH_4_Cl treatment induces MA

Treatment reduced blood pH and bicarbonate concentrations over the duration of the kinetic experiments (2-Way ANOVA, *P* < 0.0001) (Supplementary Fig. 1A,B). Chloride ion concentrations were higher in treated mice (2-Way ANOVA, *P* < 0.0001; Supplementary Fig. 1C). The pressure of carbon dioxide was lower in treated mice over the 180 days (2-Way ANOVA, *P* = 0.0027; Supplementary Fig. 1D). Blood sodium concentration was increased in treated mice (2-Way ANOVA, *P* = 0.001; Supplementary Fig. 1E). Blood potassium concentration, urea concentration and hematocrit remained unchanged following treatment (Supplementary Figs. 1F,G and H, respectively).

### Chronic NH_4_Cl treatment induces total body weight loss with an increase in energy expenditure

**Fig. 1:**
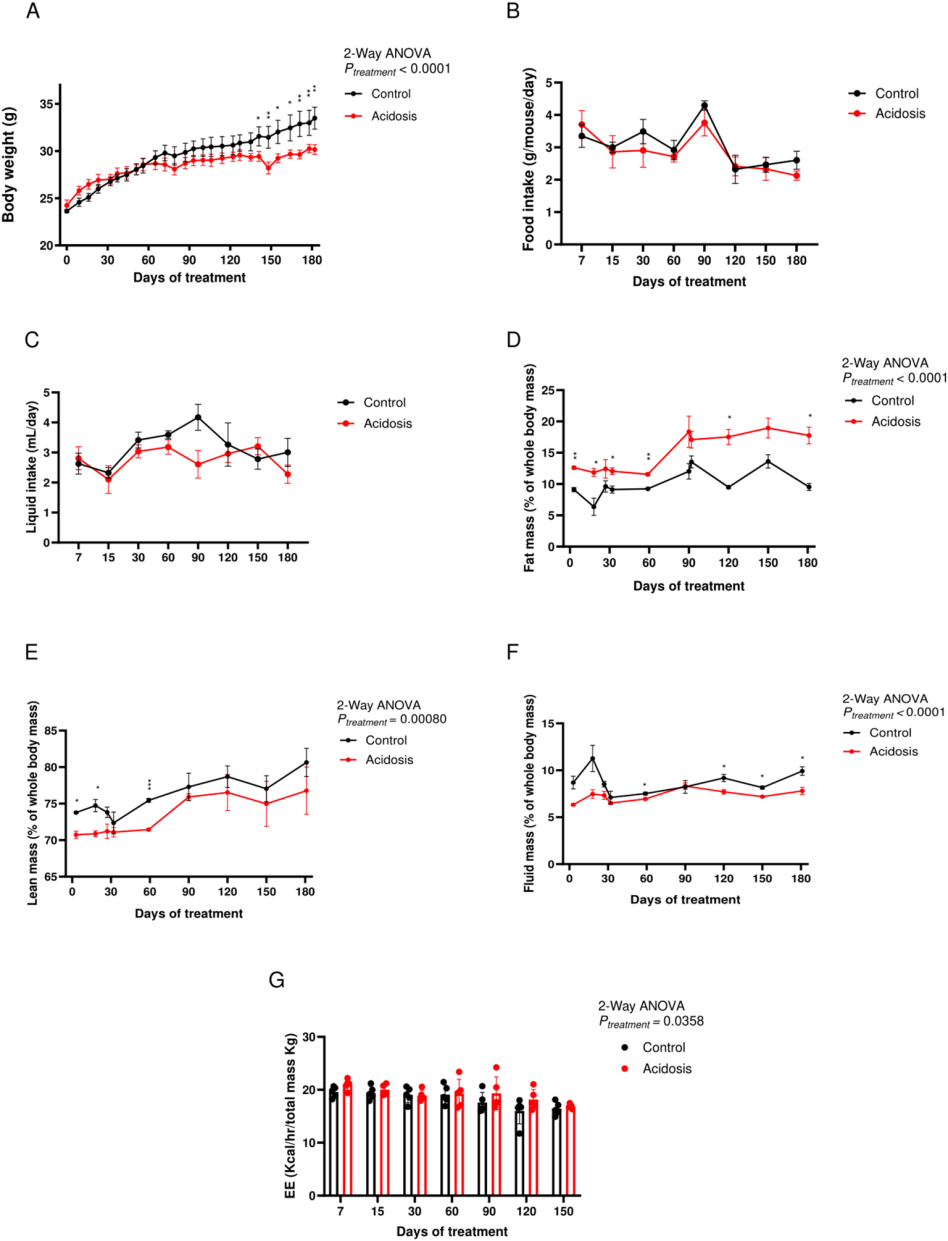
Body composition and metabolic performances are altered in acidotic mice. (**A**) Body weight of control (black) and acidotic (red) mice on chow diet between 0 and 180 days of NH_4_Cl treatment (*n* = 6 per group). (**B**,**C**) Average food (**B**) and liquid intake (**C**) of control and acidotic mice between 0 and 180 days of NH_4_Cl treatment (*n* = 6 cages per group). (**D**–**F**) Percentage of fat (**D**), lean (**E**) and fluid (**F**) mass relative to the total body weight of control and acidotic mice between 0 and 180 days of NH_4_Cl treatment (*n* = 6 for the control and 5 for the acidotic mice). (**G**) Energy expenditure of control and acidotic mice between 7 and 150 days of NH_4_Cl treatment. All values are expressed as mean ± SEM. EE: energy expenditure, ns: not significant; Kcal: kilo calories.

Control mice continuously gained weight over the duration of the study but MA mice reached a plateau after day 60 (2-Way ANOVA, *P* < 0.0001; Fig. 1A), despite having a higher food and liquid intake (2-Way ANOVA, *P* < 0.0001; Fig. 1B,C). Fat mass percentage over total body weight was higher in MA mice (2-Way ANOVA, *P* = 0.011; Fig. 1D), lean mass was lower compared to the control mice (2-Way ANOVA, *P* < 0.0001; Fig. 1E) and fluid mass was similar in both groups (Fig. 1F). Energy expenditure (EE) was overall higher in MA mice (2-Way ANOVA, *P* = 0.036; Fig. 1G).

### Chronic NH_4_Cl treatment increases glucose tolerance in mice without changes to both insulin secretion and insulin sensitivity

**Fig. 2:**
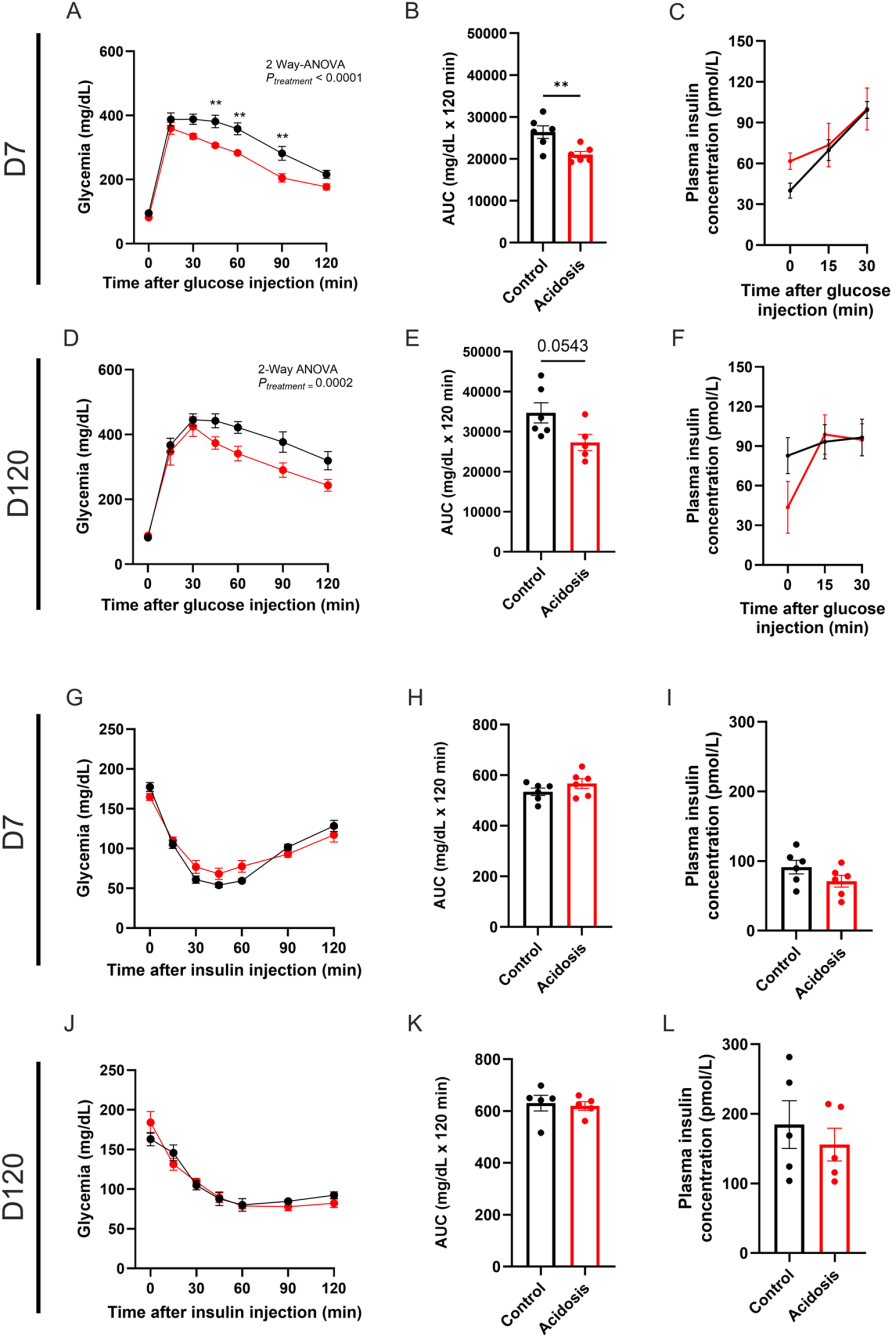
Glucose tolerance is improved without changes of insulin secretion or sensitivity in acidotic mice. (**A**–**C**) Intraperitoneal glucose tolerance test (ipGTT) in control (black) and acidotic (red) mice after 7 days of NH_4_Cl treatment (*n* = 6 per group) (**A**) with the corresponding area under the curve (AUC) with baseline values subtracted (**B**) and plasmatic insulin levels measured during ipGTT (**C**). (**D**–**F**) ipGTT in control and acidotic mice under chow diet after 120 days of NH_4_Cl treatment (*n* = 6 for the control and 5 for the acidotic mice) (**D**) and the corresponding AUC with baseline values subtracted (**E**) and plasmatic insulin levels measured during ipGTT (**F**). (**G**–**I**) Intraperitoneal insulin tolerance test (ipITT) in control and acidotic mice after 7 days of NH_4_Cl treatment (*n* = 6 per group) (**G**) and the corresponding AUC with baseline values subtracted (**H**) and basal plasmatic insulin levels measured during ipITT (**I**). (**J**–**L**) ipITT in control and acidotic mice after 120 days of NH_4_Cl treatment (*n* = 5 per group) (**J**) and the corresponding AUC with baseline values subtracted (**K**) and basal plasmatic insulin levels measured during ipITT (**L**). All values are expressed as mean ± SEM.

After 7 days of treatment, mice showed increased glucose tolerance *vs* controls (2-Way ANOVA, *P* < 0.0001; Fig. 2A). Blood glucose levels were lower in MA mice compared to controls after 45, 60 and 90 min (Šidák’s multiple comparisons test, *P* = 0.0029, *P* = 0.0025 and *P* = 0.0020 respectively; Fig. 2A). The ameliorated glucose tolerance is also observed on the area under the curve (AUC) of MA mice *vs* controls (*t*-test, *P* = 0.0097; Fig. 2B). No differences in blood insulin levels were found in MA mice when compared to controls (2-Way ANOVA, *P* = 0.33; Fig. 2C).

The improved glucose tolerance was still observed after 120 days of treatment (2-Way ANOVA, *P* = 0.0002; Fig. 2D). Basal blood glucose levels were similar in both groups (Fig. 2D), as well as 15- and 30-min post glucose injection (Fig. 2D). Glycemia of acidotic mice reduced further than control mice’s glycemia 30 min post-glucose injection (Fig. 2D). The AUC of blood glucose tended to be lower in MA mice (*t*-test, *P* = 0.054; Fig. 2E). Plasma insulin concentrations were similar in both groups (Fig. 2F).

After 15, 60, 90, 150 and 180 days of treatment, glucose tolerance was also improved in MA mice when compared to controls with no changes in insulin secretion (Supplementary Figs. 2A to 2O). Fasting glycemia of MA mice were lower compared to the controls throughout the study (2-Way ANOVA, *P* < 0.0001; Supplementary Fig. 3).

After 7 days of treatment, insulin tolerance was similar in both groups (Fig. 2G), as reflected by the respective AUCs (Fig. 2H). Fasting plasmatic insulin concentrations were comparable in both groups (Fig. 2I). Equivalent results were observed after 120 days of treatment (Fig. 2L). Fasting plasmatic insulin concentrations were alike in both groups after 15, 30, 60, 90, 150 and 180 days of treatment (Supplementary Figs. 4A to R).

### Chronic NH_4_Cl treatment alters gluconeogenesis in a tissue-specific manner

**Fig. 3:**
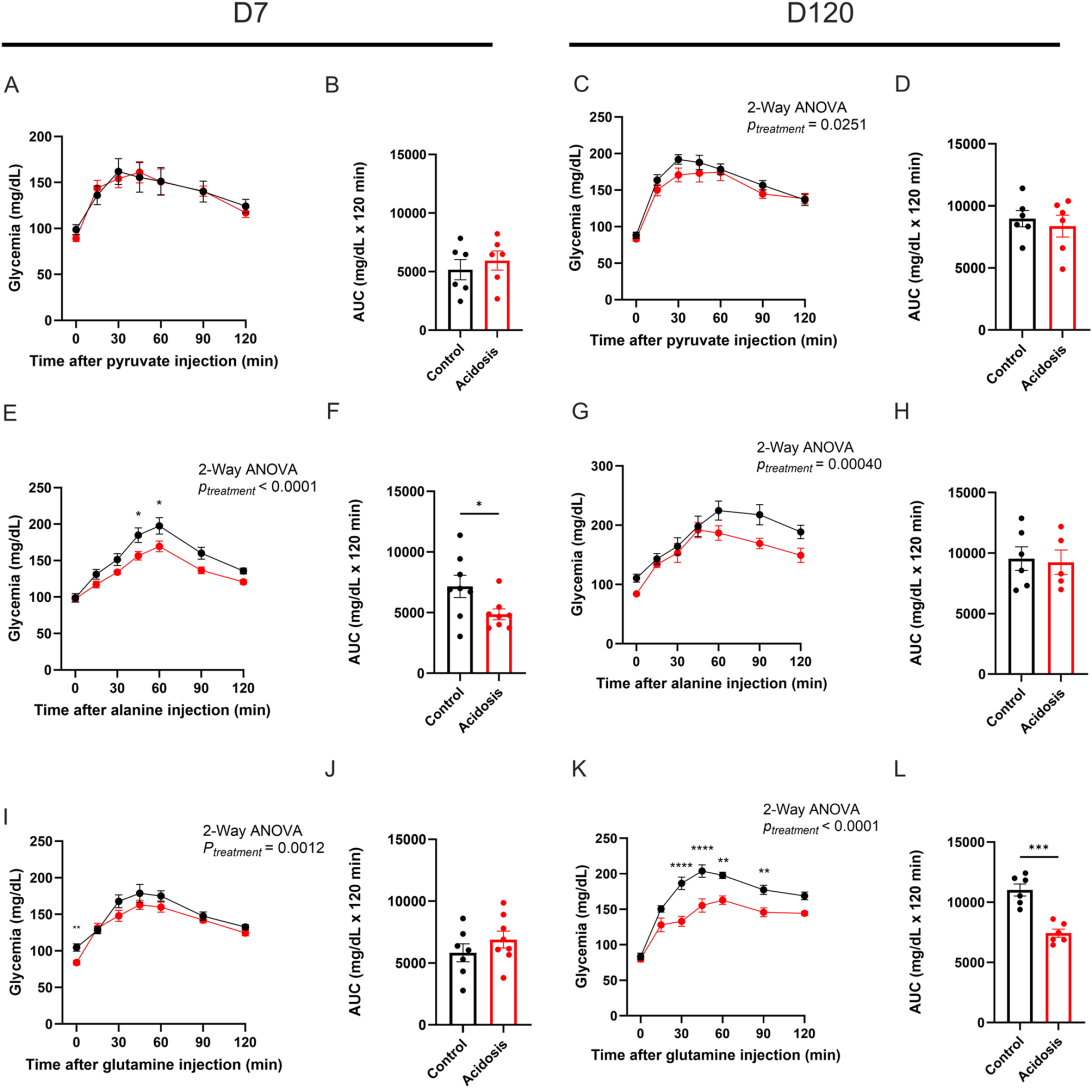
Endogenous glucose production is reduced in acidotic mice. (**A**,**B**) Intraperitoneal pyruvate tolerance test (ipPTT) (**A**) in control (black) and acidotic (red) mice after 7 days of NH_4_Cl treatment (*n* = 6 per group) with the corresponding area under the curve (AUC) of ipPTT with baseline values subtracted (**B**). (**C**,**D**) ipPTT in control and acidotic mice after 120 days of NH_4_Cl treatment (*n* = 6 per group) (**C**) and the AUC with baseline values subtracted (**D**) of ipPTT. (**E**,**F**) Intraperitoneal alanine tolerance test (ipAlaTT) (**E**) in control (black) and acidotic (red) mice after 7 days of NH_4_Cl treatment (*n* = 8 per group) and the corresponding AUC with baseline values subtracted (**F**). (**G**,**H**) IpAlaTT in control and acidotic mice under chow diet after 120 days of NH_4_Cl treatment (*n* = 6 for the control and 5 for the acidotic mice) (**G**) and the corresponding AUC with baseline values subtracted (**H**). (**I**,**J**) Intraperitoneal glutamine tolerance test (ipGluTT) in control (black) and acidotic (red) mice under chow diet after 7 days of NH_4_Cl treatment (*n* = 7 for the control and 8 for the acidotic mice) (**I**) and the corresponding AUC with baseline values subtracted (**J**). (**K**,**L**) ipGluTT in control and acidotic mice under chow diet after 120 days of NH_4_Cl treatment (*n* = 6 per group) (**K**) with the AUC of ipPTT (**L**). All values are expressed as mean ± SEM.

After 7 days of treatment, pyruvate induced endogenous glucose production (EGP) was similar in both groups (Fig. 3A) as shown in the corresponding AUC (Fig. 3B). Comparable results were observed after 15 (Supplementary Figs. 5A–C) and 30 days of treatment (Supplementary Figs. 5D–F). However, 60 days of treatment led to reduced gluconeogenesis *vs* controls (2-Way ANOVA, *P* = 0.00030; Supplementary Fig. 5G). This effect was even more pronounced after 90 days of treatment (2-Way ANOVA, *P* < 0.0001; Supplementary Fig. 5 J). Blood glucose was lower in MA mice at 30 (*t*-test, *P* = 0.022 Supplementary Fig. 5 J) and 60 min post-pyruvate injection (*t*-test, *P* = 0.025 Supplementary Fig. 5 J), without differences in the AUC (Supplementary Fig. 5 K). After 120 days of treatment, total gluconeogenesis was reduced in MA mice *vs* controls (2-Way ANOVA, *P* = 0.025; Fig. 3C), without changes of the AUC (Fig. 3D). After 150 and 180 days of treatment, no differences were observed between both groups (Supplementary Figs. 5 M to R). No differences in insulin secretion were observed during the PTTs (data not shown).

Then, alanine (hepatic EGP substrate) and glutamine (renal and intestinal EGP substrate) stimulated gluconeogenesis tests were performed. MA mice had reduced hepatic EGP after 7 days (2-Way ANOVA, *P* < 0.0001; Fig. 3E), confirmed by the AUCs (*t*-test, *P* = 0.041; Fig. 3F). Hepatic EGP was also reduced in treated mice after 120 days (2-Way ANOVA, *P* = 0.00040; Fig. 3G), with no differences in the AUC (Fig. 3H) Reduced hepatic gluconeogenesis was not or barely observable after 30, 60 and 90 days of treatment (Supplementary Figs. 6). Chronic treatment also led to reduced renal/intestinal gluconeogenesis. From day 7 of the study, MA mice produced less glucose after glutamine injection *vs* controls (2-Way-ANOVA, *P* = 0.0012; Fig. 3I), without changes of the AUC (Fig. 3J). This was also the case after 30 and 60 days of treatment (2-Way ANOVA, *P* < 0.0001; Supplementary Figs. 7A,C). No effects were observed at 90 days of treatment between both groups (Supplementary Figs. 7E,F). However, 120 days of treatment led to a reduction of glutamine stimulated EGP *vs* controls (2-Way ANOVA, *P* < 0.0001; Fig. 3K). Glutamine derived EGP was lower in acidotic mice after 30-, 45-, 60- and 90-min post injection (Šidák’s multiple comparison test, P < 0.0001, P < 0.0001, P = 0.0030 and P = 0.0092 respectively; Fig. 3K). The AUC of glycemia in MA mice was lower *vs* controls (*t*-test, *P* = 0.0010; Fig. 3L).

### Chronic NH_4_Cl treatment increases the expression of key actors regulating renal and intestinal gluconeogenesis and decreases the expression of those involved in hepatic gluconeogenesis

**Fig. 4:**
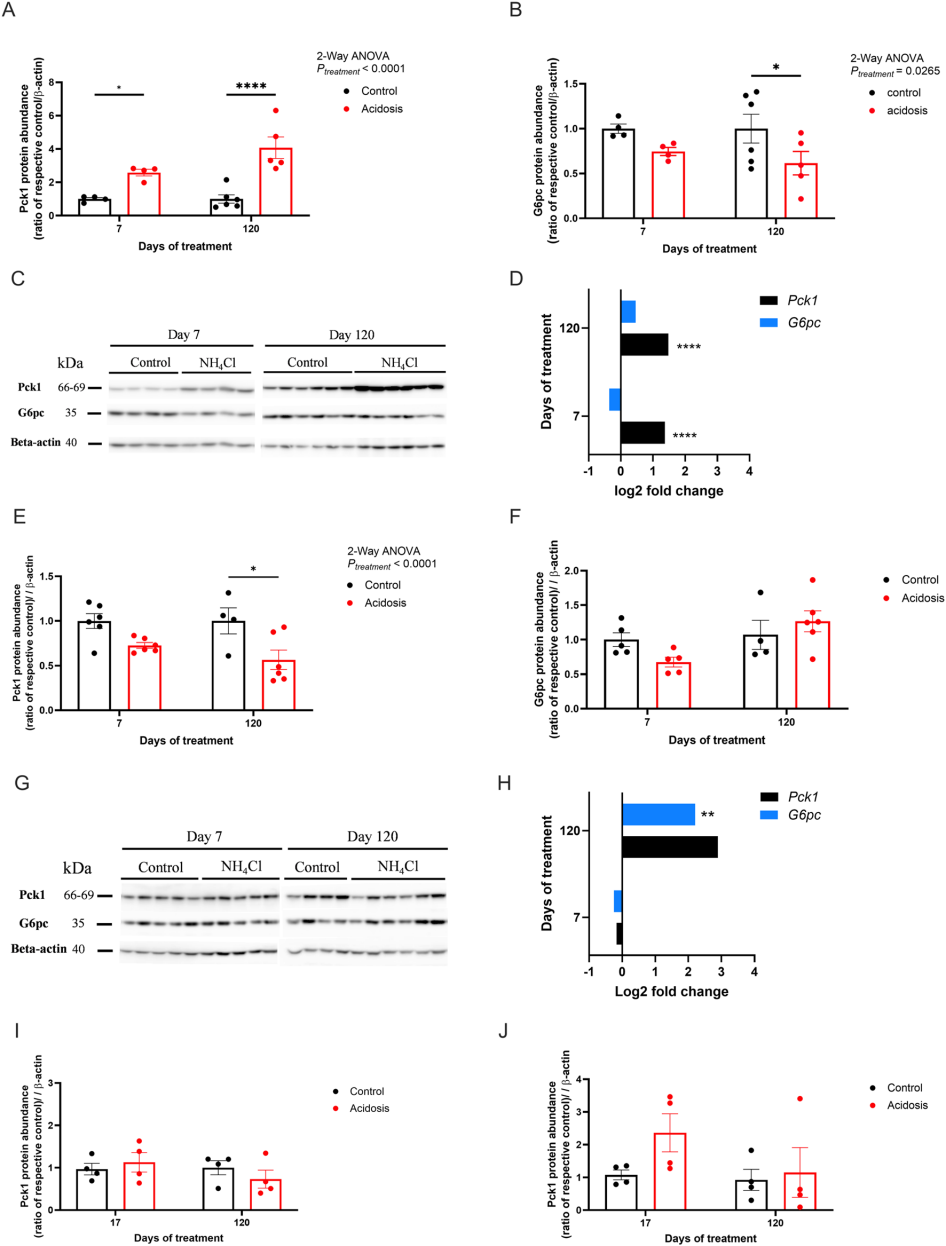
The expression of key actors of hepatic and extra hepatic gluconeogenesis is altered in a tissue specific manner in acidotic mice. (**A**–**C**) Quantification of Pck1 (**A**) and G6pc (**B**) from a Western Blot assay (**C**) showing protein levels in kidney lysates of control (black) and acidotic (red) mice (*n* = 4 per group at day 7 and 6 at day 120) Beta-actin was used as a loading control. Quantification was performed using the ImageQuant software. (**D**) Log2 fold change of mRNA expression of *Pck1* and *G6pc* in the kidney of control and acidotic mice after 7 and 120 days of treatment (*n* = 4 per group). (E–G) Quantification of Pck1 (**E**) and G6pc (**F**) from a Western Blot assay (**G**) showing protein levels in liver lysates of control (black) and acidotic (red) mice (*n* = 5 at day 7 and 4 for the control and 6 for the NH_4_Cl at day 120) Beta-actin was used as a loading control. Quantification was performed using the ImageQuant software. (**H**) Log2 fold change of mRNA expression of *Pck1* and *G6pc* in the liver of control and acidotic mice after 7 and 120 days of treatment (*n* = 4 per group). (**I**,**J**) Quantification of PCK1 in the duodenum (**J**) and jejunum (**K**) from a Western Blot assay showing protein levels of control and acidotic mice after 17 and 120 days of treatment (*n* = 4 per group); beta-actin was used as a loading control. All blots have been cropped for presentation purposes and original blots are presented in Supplementary Figs. 11 and 12. Quantification was performed using the ImageQuant software. All values are expressed as mean ± SEM except for the Log2 fold change values.

To analyze further the effects of chronic MA on tissue specific gluconeogenesis, protein and RNA expression of Pck1/*Pck1* and G6pc/*G6pc* were assessed in the kidney, the liver, the duodenum and the jejunum^25^. Renal Pck1 protein expression was increased by the treatment (2-Way ANOVA, *P* < 0.0001; Fig. 4A,C and Supplementary Fig. 11) whereas G6pc expression was reduced only in the later stages of the kinetic study (2-Way ANOVA, *P* = 0.026; 120 days of treatment* P* = 0.038; Fig. 4B,C and Supplementary Fig. 11). RNA expression analysis showed a marked upregulation on renal *Pck1* throughout the study (Fig. 4D) and no significant difference of *G6pc* renal expression (Fig. 4D). Hepatic Pck1 expression reduced at the latter stages of the study (2-Way ANOVA, *P* < 0.0001; 120 days of treatment *P* = 0.012; Fig. 4E and Supplementary Fig. 12), but no differences were observed at the early stages of the study (Fig. 4E,G and Supplementary Fig. 12). Chronic MA did not alter hepatic G6pc expression (Fig. 4F,G and Supplementary Fig. 12). mRNA expression of both *Pck1* and *G6pc* was increased in the liver in treated animals compared to their controls (2-Way ANOVA, *P* < 0.0001 and *P* = 0.00090 respectively; Fig. 4H). Duodenunal and jejunal Pck1 expression were not altered after 17 and 120 days of treatment (Fig. 4I,J). RNA-seq showed that the renal expression of *Pcx* (pyruvate carboxylase), *Pck1* (phosphoenolpyruvate carboxykinase 1, cytosolic), *Fbp1* (fructose-bisphosphatase 1), *Fbp2* (fructose-bisphosphatase 2) and *G6pc* (glucose-6-phosphatase) was altered between 3 and 60 days of treatment (Supplementary Fig. 8).

### Increased glucose urinary excretion is associated with lower expression of renal sodium/glucose co-transporters in acidotic mice

**Fig. 5:**
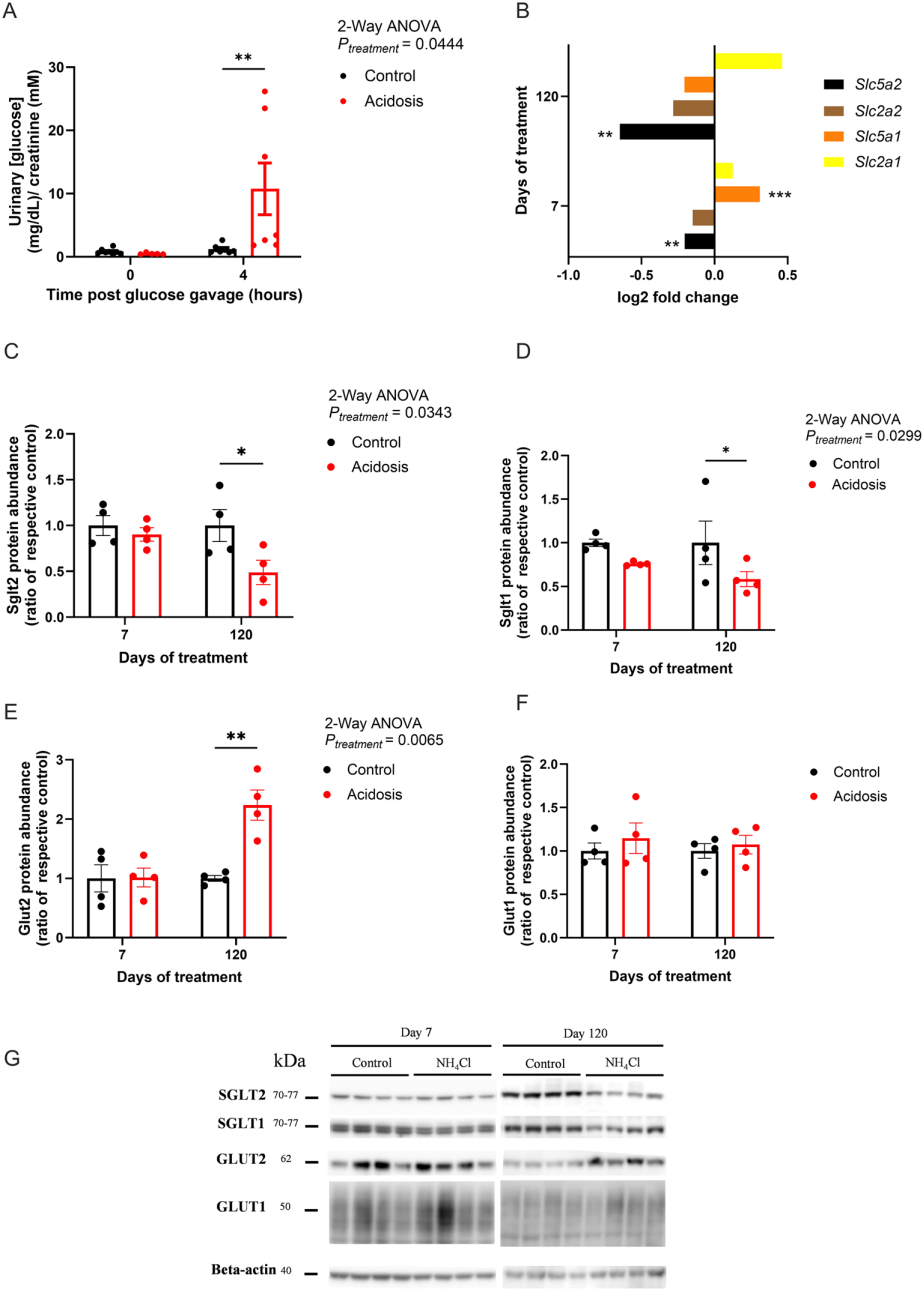
Increased glucose urinary excretion is associated with lower expression of renal sodium/glucose co-transporters in acidotic mice. (**A**) Urinary glucose concentration before and after an oral bolus of 2 g/kg of body weight in control mice (black) and mice treated for 90 days (red) (T0, *n* = 8 for the control and 5 for the acidotic mice. T4, *n* = 7 per group). (**B**) Log2 fold change of mRNA expression of *Slc5a2*, *Slc5a1*, *Slc2a2*, and *Slc2a1* in the kidney of control and acidotic mice after 7 and 120 days of treatment (*n* = 4 per group). (**C**–**G**) Quantification of Sglt2 (**C**), Sglt1 (**D**), Glut2 (**E**) and Glut1 (**F**) from a Western Blot assay (**G**) showing protein levels in kidney of control (black) and acidotic (red) mice (*n* = 4 per group). Beta-actin was used as a loading control. All blots have been cropped for presentation purposes and original blots are presented in Supplementary Fig. 13. Quantification was performed using the ImageQuant software. (**G**) All values are expressed as mean ± SEM except for the Log2 fold change values.

Glucose homeostasis is regulated by endogenous glucose production/breakdown, glucose renal clearance and glucose uptake/storage^24–26^. We therefore assessed renal excretion of glucose in MA mice by measuring glucose excretion 4 h after an oral glucose bolus. MA mice showed an increase in glucosuria *vs* control mice after 7, 30 (Sidak’s multiple comparison test, *P* = 0.031 and *P* = 0.035 respectively; data not shown), and 90 days of treatment (Sidak’s multiple comparison test, *P* = 0.0076; Fig. 5A).

The expression of renal glucose transporters, located in the proximal tubule, was therefore investigated. *Slc5a2 (Sglt2)* mRNA of treated mice was downregulated compared to control mice after short and long terms of the kinetic study (J7, P = 0.0098; J120, P = 0.0013, Fig. 5B). Expression of *Slc5a1 (Sglt1)* mRNA was upregulated in acidotic mice compared to their control at short terms of the kinetic study (J7, P = 0.00053, Fig. 5B). mRNA expression of both *Slc2a2 (Glut2)* and *Slc2a1 (Glut1)* were not altered by the treatment (Fig. 5B).

Protein expression of both sodium glucose co-transporters were not altered by the NH_4_Cl load after 7 days, but were significantly reduced over 120 days (Sidak’s multiple comparison test, *P* = 0.030 and *P* = 0.047; Fig. 5C,D respectively and Fig. 5G and Supplementary Fig. 13). Glut2 expression was not altered by the treatment after 7 days, but its protein expression was significantly upregulated after 120 days (Sidak’s multiple comparison test, *P* = 0.0012, Fig. 5E,G and Supplementary Fig. 13). Glut1 renal expression was not altered by NH_4_Cl load (Fig. 5F,G and Supplementary Fig. 13).

### Chronic MA does not alter whole body 2-deoxy-2-[18F] fluoroglucose uptake but increases glucose uptake in kidney and bladder

**Fig. 6:**
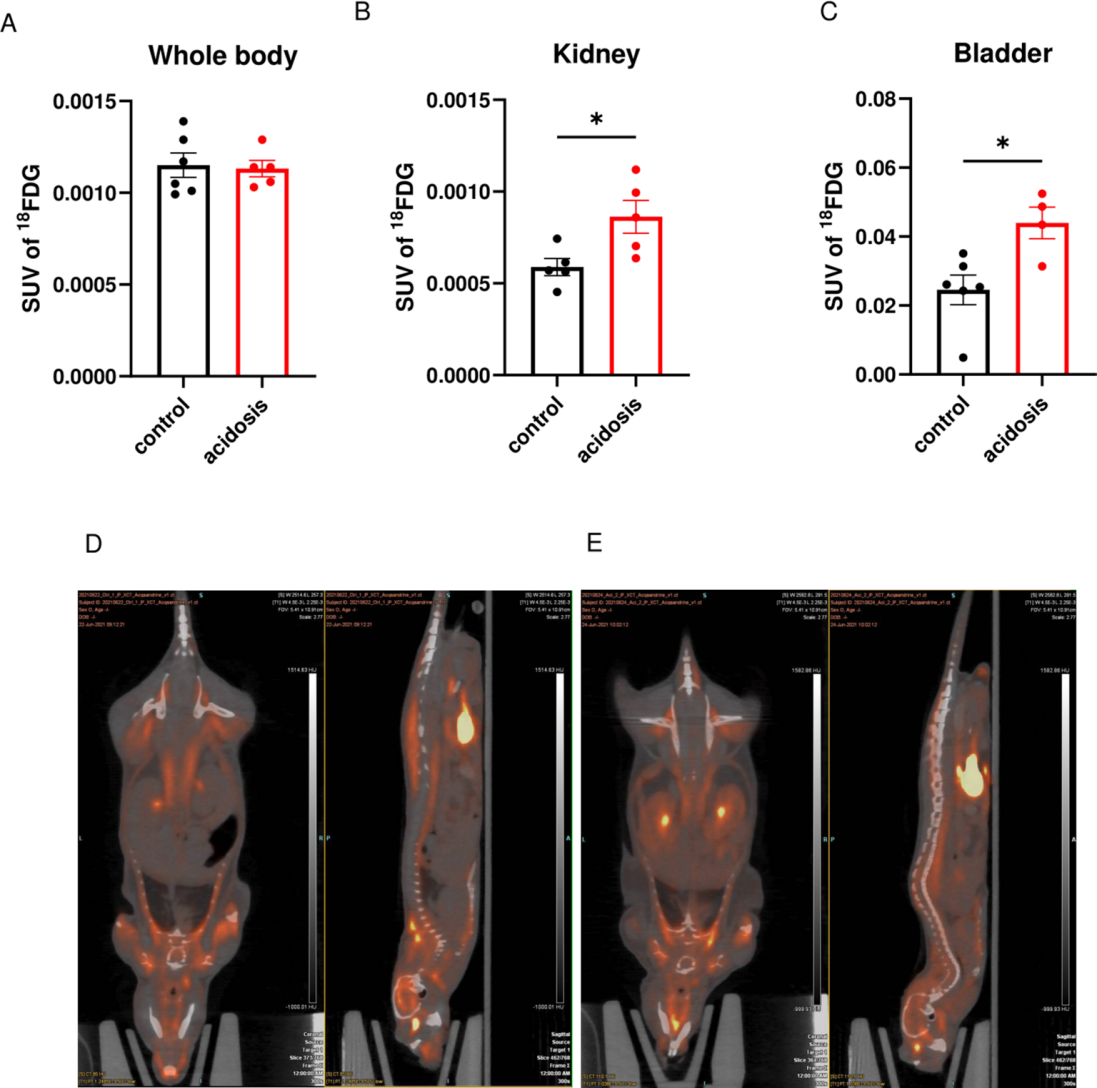
180 days of NH_4_Cl treatment does not alter whole body 2-Deoxy-2-[18F]fluoroglucose uptake but increases glucose uptake in kidney and bladder. (**A**–**C**) Standard uptake values (SUV) of 2-Deoxy-2-[18F]fluoroglucose (^18^FDG) in the whole body (**A**), kidney (**B**) and bladder (**C**) of control (black) and acidotic (red) mice under chow diet after 180 days of NH_4_Cl treatment (*n* = 6 for the control and 5 for the acidotic mice). (**D**,**E**) Positron emission tomography (PET) scan of a control (**D**) and acidotic mouse (**E**) after injection of ^18^FDG. All values are expressed as mean ± SEM. *SUV* standard uptake value.

Following the 2-Deoxy-2-[18F] fluoroglucose (^18^FDG) injection after 180 days of NH_4_Cl load, whole-body standard uptake value (SUV) was similar in both groups (Fig. 6A). This was also observed at the tissue level in muscle, inguinal white adipose tissue, perigonadal white adipose tissue, brown adipose tissue and brain (Supplementary Figs. 9A to E). However, MA mice displayed an increase in ^18^FDG SUV in the kidney (~ 1.5-fold) and bladder (~ twofold) (unpaired *t*-test, *P* = 0.026 and* P* = 0.017, respectively; Fig. 6B–E).

### Chronic MA potentiates anion transport, glucose and lipid metabolism, mitochondrial and oxidative phosphorylation pathways in the kidney

**Fig. 7:**
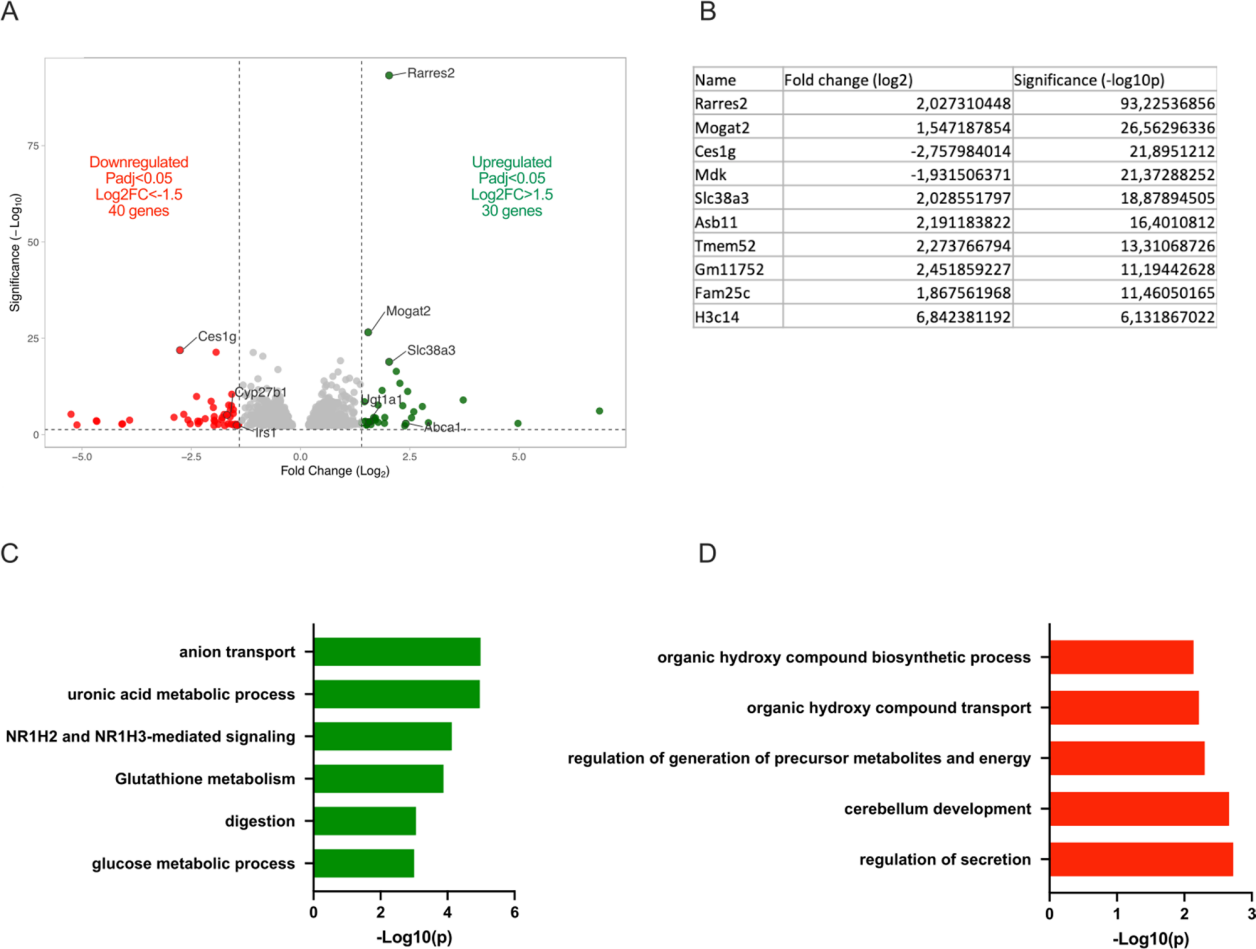
Transcriptomic analysis in acidotic compared to control mice after 7 days of NH_4_Cl treatment. (**A**) Volcano plot of the genes up- and down regulated by the treatment. (**B**) List of top 10 genes altered by metabolic acidosis. Functional enrichment of genes (**C**) upregulated and (**D**) downregulated by 7 days of NH_4_Cl. Summary pathways analyzed by Metascape − *log*10(p) values greater than 1,5 and less than − 1,5 were selected with a False Discovery Rate (FDR) less than 0.05 and a *p* value less than 0.05.

To decipher altered mechanisms in response to chronic MA, transcriptomic analysis of whole kidney was determined by RNA-seq between 3 and 60 days of treatment (Supplementary Table 3 and Supplementary Fig. 10). 70 genes were differentially expressed (30 up-regulated and 40 down-regulated) after 7 days of treatment (Fig. 7A). Sequencing analysis revealed a strong enrichment of upregulated genes in the anion transport pathway (such as *Best1, Slc13a2, Slc38a3, Slc13a4, Slc26a6, Slc25a25, Abca17, Lcn2, Fabp5* and *Cox6a2;* Fig. 7C and Supplementary Table 1). A marked activation of glucuronic metabolism (i.e., up-regulation of *Dcxr, Ugt1a1, Ugt1a3, Gstm5, Nat8, Rarres2, Pck1, Ptgds, Bace2* and *Abca1*; Fig. 7B,C and Supplementary Table 1) and glucose metabolism was observed (i.e., up-regulation of *Fabp5, Pck1, Dcxr, Abca1, Ptgds, Mogat2, Fitm1, Phospho1 and Bace2*; Fig. 7B,C and Supplementary Table 1). NH_4_Cl loading had an inhibitory effect on renal secretion regulation (i.e., down-regulation of *Ces1g, Cyp27b1, Irs1, Septin2, Syt7, Exph5, Zbed6* and *Prr5l;* Fig. 7B,D and Supplementary Table 1).

**Fig. 8:**
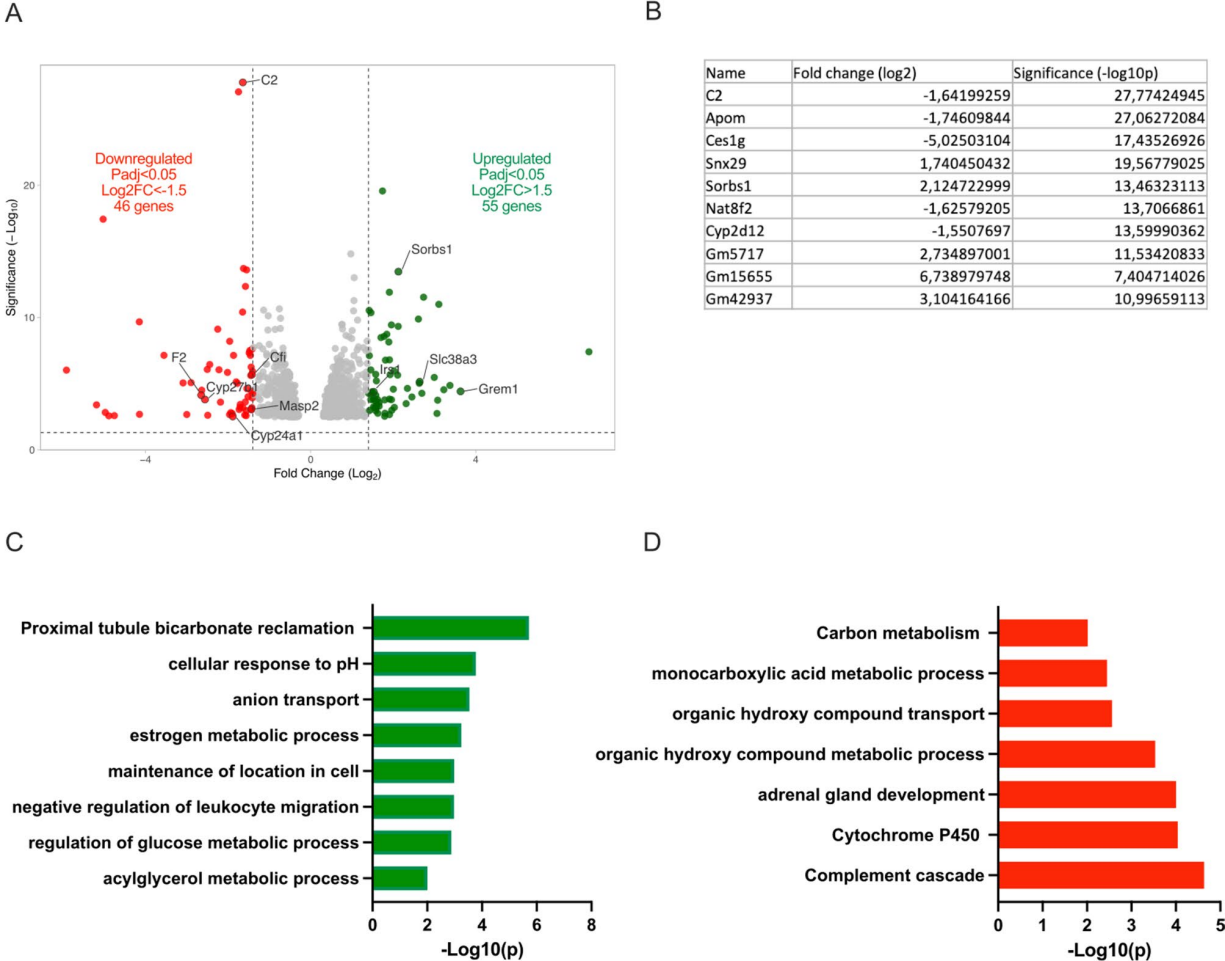
Transcriptomic analysis in acidotic compared to control mice after 60 days of NH_4_Cl treatment. (**A**) Volcano plot of the genes up- and down regulated by the treatment. (**B**) List of top 10 genes altered by metabolic acidosis. Functional enrichment of genes (**C**) upregulated and (**D**) downregulated by 60 days of NH_4_Cl. Summary pathways analyzed by Metascape -*log*10(p) values greater than 1,5 and less than − 1,5 were selected with a False Discovery Rate (FDR) less than 0.05 and a *p* value less than 0.05.

101 genes were differentially expressed (55 up-regulated and 46 down-regulated; Fig. 8A) after 60 days of treatment. Proximal tubule bicarbonate reclamation, cellular response to pH and anion transport were still upregulated in treated mice (i.e., up-regulation of *Gls, Glud1, Pck1, Slc38a3, Slc34a2, Slc16a14, Slc16a6, Slc26a7, Slc26a10 Slc4a7* and *Slc10a5*; Fig. 8B,C and Supplementary Table 1). Activation of the negative regulation of leukocyte migration (i.e., up-regulation of *Grem1, Ccl28, Mmp28, Atp7a, Loxl4* and *Papln*; Fig. 8C and Supplementary Table 1) and increased regulation of glucose metabolism (i.e., up-regulation of *Irs1, Sorbs1, Dgkq* and *Pdk1;* Fig. 8A–C and Supplementary Table 1) were also observed. Chronic NH_4_Cl loading inhibited the complement cascade (i.e., down-regulation of *C2, Cfi, F2, Masp2* and *Igkc*; Fig. 8A,B,D and Supplementary Table 1) and downregulated genes involved in the cytochrome P450 mechanism (i.e., *Cyp24a1, Cyp27b1, Cyp4b1, Tbxas1, Inmt, Miox* and *Slc34a3*; Fig. 8A,B,D and Supplementary Table 1).

## Discussion

Acute MA has been linked to glucose intolerance via insulin resistance in both animals and humans^14–19^. In this study, chronic MA by NH₄Cl loading produced marked and sustained effects on energy metabolism, glucose homeostasis, and renal function. The reduction in blood pH, bicarbonate concentration accompanied by elevated plasma chloride, confirm the establishment of chronic MA. These biochemical changes are in line with previous observations in NH₄Cl-loaded models and validate the use of this approach to mimic acidotic states in vivo^27–29^. A striking outcome of chronic MA was the divergence between similar food and drink intake and the attenuation of body weight gain. Despite similar energy intake, treated mice displayed increased energy expenditure, reduced lean mass, and increased fat distribution. These results suggest that acidosis imposes a metabolic cost that impairs anabolic processes, as protein breakdown is accelerated to supply renal ammoniagenesis with substrates such as glutamine^20^.

Strikingly, chronic MA improved glucose tolerance, without altering insulin secretion or overall sensitivity, suggesting enhanced non–insulin-mediated glucose disposal, previously reported by Mannon et al*.*^30^ in Sprague–Dawley rats with a 7 days NH_4_Cl load. It is important to note that, as mentioned earlier, acidotic mice displayed a reduced lean mass compared to controls and therefore, could have a tissue dependent effect on insulin sensitivity. Although this is the case, contrary to humans, fat mass is highly metabolically active in mice and is a substantial contributor of total glucose disposal and insulin dosage has been adjusted to total body weight as described in section “Animals experiments”. Further investigation on the tissue specific insulin sensitivity via the activation of its pathway would further deepen our understanding on the matter. Several mechanisms may underlie this: (i) reduced hepatic and intestinal gluconeogenesis, (ii) glucosuria secondary to downregulation of SGLT’s and (iii), increased renal glucose metabolism evidenced by elevated FDG uptake in the kidney and transcriptomic analysis. These findings challenge the classical view of acidosis as deleterious for glucose metabolism and suggest context-dependent benefits. Indeed, the mechanisms linking acute MA and alterations in insulin activity remain elusive. It is suggested that the acid-mediated inhibiting effects on insulin binding affinity to its receptor, on glycolysis, on the recycling of glucose transporters, and on insulin secretion may account for this phenomenon ^19–24,31–33^. In previous experiments, we found that treated mice who were given NH_4_Cl for 16 h had similar glucose tolerance compared to their control (data not shown) whereas an increase was observed after 3 days and until 180 days. Data therefore suggest a biphasic response to acidosis between acute and chronic treatments.

Further mechanistic insights are provided as we showed that chronic MA regulates gluconeogenesis in a tissue-specific manner, resulting in a global reduction in fasting glucose. Total EGP induced by pyruvate was unchanged after 7 days, but was reduced in treated mice after 120 days, reinforcing the metabolic shift hypothesis. Reduced alanine stimulated EGP after 7 and 120 days suggests that chronic MA inhibits the hepatic gluconeogenic pathway, confirmed by the reduction Pck1 abundance observed in the liver^33,34^. Acidosis has been shown to reduce the hepatic oxaloacetate (OAA) to malate ratio, limiting substrate availability for conversion to P-enolpyruvate by Pck1^33^. Increased H⁺ and NADH during acidosis may favor malate accumulation and OAA depletion via malate dehydrogenase 2^2,35,36^. Chronic MA can therefore modulate hepatic TCA cycle flux and tissue specific EGP. Paradoxically, hepatic *Pck1* knockout has been reported to increase total TCA flux, potentially contributing to the improved glucose tolerance observed in MA mice^37,38^. These findings suggest that chronic MA downregulates hepatic gluconeogenesis by reducing Pck1 expression, potentially caused by an altered OAA/malate balance.

It is known that MA enhances glutamine metabolism by simultaneously generating glucose and ammonia to support systemic glucose supply while facilitating acid excretion^39^. Renal Pck1 expression was consistently upregulated while G6pc was downregulated at later stages, suggesting that Pck1 expression during MA is crucial to drive EGP production^25^. We can therefore assume that the reduced glutamine induced EGP throughout our study is mainly due to a reduced duodenal and/or jejunal EGP. Indeed, glutamine fuels EGP in the kidney and small intestine (duodenum and jejunum), where the enzymatic machinery for glutamine catabolism is abundant, whereas the liver lacks glutaminase activity and instead relies predominantly on alanine and lactate^40^. Although jejunal Pck1 protein abundance was unchanged in treated mice, G6pc seems to plays a more decisive role in driving EGP in this tissue^41^.

It is important to keep in mind that glycogenolysis was not discussed in this paper, despite being an important part of EGP. Although this is the case, all pyruvate, glutamine and alanine tests have been done after 16 h fasts, which have shown to deplete almost entirely hepatic glycogen stores^42,43^, showing a shift from glycogenolysis to gluconeogenesis over longer periods of fast. Further analysis on glycogenolysis and glycogen content would be needed to further deepen our understanding of the mechanisms by which acidosis improves glucose tolerance.

In parallel, MA reduced renal glucose reabsorption by downregulating *Sglt2* and *Sglt1* expression and reducing their protein abundance at later stages^44^. This is not due to kidney damage as although glomerular filtration rate (GFR) was not measured, plasma creatinine concentration was similar between both groups and no histological alterations were observed (data not shown). Additionally, either a strong decrease in GFR or hyperglycemia could account for the glucosuria observed as early as 7 days after NH_4_Cl loading, and neither were observed^45,46^. RNA-seq revealed downregulation of *Hnf1a*, *Hnf4a*, and *NF-κB (*via ubiquitin-dependent degradation of cyclin D^47,48^), key transcriptional regulators of *Sglt1* and *Sglt2*^49–54^, likely contributing to their reduced expression. These changes may confer renoprotection, as SGLT2 inhibition reduces ROS-related enzymes, including Nox2, Nox4, TGF-β1, MCP-1, ICAM-1, and MPO^55–57^, many of which were downregulated in NH₄Cl-treated kidneys. This was associated with significant glucosuria, a phenomenon reminiscent of pharmacological SGLT2 inhibition. Interestingly, Glut2 expression was increased at later stages of the kinetic study, potentially as a compensatory mechanism, to facilitate basolateral glucose export. These adaptations emphasize the kidney’s central role in integrating glucose homeostasis with acid–base regulation. The glucosuria observed was measured prior and following a glucose oral bolus of 2 g/kg to challenge the renal glucose handling. This could be considered as a limitation as urine was not collected over 24 h in metabolic cages. Although this is the case, the aim of this experiment was to quantify the excretion of glucose with elevated glycemia to analyze if acidotic mice had a lower reabsorption threshold than their control, which is the case.

Transcriptomic analysis revealed broader metabolic adaptations. Genes involved in anion transport, bicarbonate reclamation, mitochondrial oxidative phosphorylation, and glucose and lipid metabolism were upregulated, consistent with renal metabolic reprogramming to support ammoniagenesis and maintain cellular energy^58–66^. Functional studies are required to validate transcriptomic findings, as mRNA expression does not always reflect protein abundance^67^. Time-course analysis demonstrated a biphasic transcriptional response: 156 genes were altered at day 3, with the number halving by day 7 and remaining stable thereafter, consistent with a transition from acute to chronic adaptation^68^.

NH_4_Cl supplementation has been classically used to induce MA in rodents, dogs and humans^14–16,18,21,28,68^. Although the dehydration effects of NH_4_Cl treatment remain controversial, we did not observe it in our study. Indeed, genes involved in osmotic regulation and water homeostasis such as aquaporin 2 and 3, the angiotensin 2 receptor, gremlin 2, the urea transporter, the vasopressin 2 receptor^28,68,69^ are not modified in the kidney of treated mice (data not shown). Additionally, male mice were used exclusively in this study to reduce variability associated with the estrous cycle in female mice, which can influence hormonal levels and potentially confound experimental outcomes.

## Conclusion

Overall, our data show that, unlike acute acid–base disturbance, chronic MA improves glucose tolerance without changes in insulin sensitivity, likely due to reduced hepatic gluconeogenesis, decreased renal glucose reabsorption and increased energy demands in the kidney.

## Material and methods

### Ethical statements

Seven-week old male C57Bl/6JRj mice were purchased from Janvier labs (Mayenne. France). *In vivo* experiments at Inserm 1283 – EGID, Université de Lille, Lille (France) were performed in compliance with the EU Directive 2010/63/EU for animal experimentsAnimal house agreements no. B 59-35010 (Authorization for Animal Experimentation no. 2020020516511947, Project approval by our local ethical committee no. CEEA 23998) and *in vivo* experiments at Inserm 1188 – Université de la Réunion, CYROI, Saint Clotilde, Reunion Island (France) were performed in compliance with the EU Directive 2010/63/EU for animal experimentsAnimal house agreements no. A 974 001 (Authorization for Animal Experimentation no. 201806111409218, Project approval by our local ethical committee no. CEEA 114). All methods are carried out in accordance with relevant guidelines and regulations and are reported in accordance with ARRIVE guidelines. At the time of euthanasia, the weight range of the mice was 28-40 g. All animals were euthanized either by cervical dislocation or cardiac puncture with Buprenorphine (0.05 mg/kg s.c.) and 4% isoflurane with a O_2_ flux of 1 L/min for the initial anesthesia and 2% isoflurane with a O_2_ flux of 0.5 L/min to maintain the anesthesia. All efforts were made to minimize suffering.

### Animals experiments

Following the 10-day acclimatization, MA mice were given 0.28 M NH_4_Cl (Sigma) in their sterile distilled drinking water. Control mice were given sterile distilled drinking water. Each mouse, as well as their food and liquid consumption, were weighted weekly. Experimental procedures took place after 3, 7, 14, 30, 60, 90, 150 and 180 days of NH_4_Cl treatment. Metabolic phenotyping was performed as previously described^25,70^. Briefly, intraperitoneal glucose (2 g of glucose per kg of body weight), insulin (0.75 U of insulin per kg of body weight), pyruvate (targeting global gluconeogenesis; 1 g of pyruvate per kg of body weight), alanine (targeting hepatic gluconeogenesis; 1 g of alanine per kg of body weight) and glutamine (targeting extra-hepatic gluconeogenesis; 1 g of glutamine per kg of body weight) tolerance tests were performed. All tests were done after a 16-h fast, except the ipITT which were done after a 6-h fast.

Glycemia was measured before and at different time after injections using the Accu-Check Performa (Roche Diagnostics) glucometer. Plasma insulin levels were measured using the mouse Insulin Elisa kit (Mercodia). Metabolic rate was measured by indirect calorimetry using the Phenomaster metabolic cage system (TSE Systems). Mice were housed individually and maintained at 21 °C under a 12 h light/12 h dark cycle. Food and water were available ad libitum. Lean, fat and fluid mass were measured using a Minispec LF50 (Bruker).

### Sample analysis

Blood chemistry was measured using i-STAT EC8 + cartridge and i-STAT1 analyser (Abbott) on anesthetized animals using Buprenorphine (0.05 mg/kg s.c.) and 4% isoflurane with a O_2_ flux of 1 L/min. Blood samples were taken by retro-orbital harvesting and used fresh at the time of the experiment. For urinary glucose concentration quantifications, mice were orally given 2 g/kg of glucose and urine collection was performed at T0 and T4h. Quantifications were done using a mouse glucose assay (ref: 81,692; Chrystal Chem) following manufacturer’s instructions and normalized with creatinuria concentration. Urinary creatinine concentration was measured by ionic chromatography and spectrophotometry (235 nm).

### Protein extracts and immunoblot analysis

Protein extraction and Western blots were carried out as previously described^71^. Tissues lysis was performed by using 150 mM NaCl, 1% Triton X-100; 50 mM Tris pH 7.8 and phosphatase (Roche) and protease inhibitors (Pierce) on ice. The renal plasma membrane-enriched fractions were prepared by differential centrifugation in 250 mM sucrose, 100 mM Tris-Hepes at pH 7.4 and phosphatase (Roche) and protease inhibitors (Pierce). Western blotting was performed using 15 μg for the kidney, liver and intestine lysates and 22.5 μg for the plasma membrane-enriched fractions of proteins loaded on SDS-PAGE precast gel (Biorad). The list of antibodies and the concentrations used are listed in Supplementary Table 1.

### RNA expression and sequencing (RNA-seq)

Kidney and liver total RNA were extracted with the Rneasy lipid tissue (Qiagen) mini kit following manufacturer’s instruction. qRT-PCR was carried out as previously described^70^. Forward and reverse primers used are listed in Supplementary Table 2. RNA sequencing was done using the Kapa mRNA Hyperprep kit (Roche) in combination with the HiSeq 4000 sequencing system (Illumina). The demultiplexing of sequence data was performed using bcl2fastq Conversion Software (Illumina; bcl2fastq v2.20.0). Trimming of adapter sequences and low-quality reads was performed using trimmomatic software (version 0.39). Reads quality control was assessed using FastQC (v0.11.9). Subsequently, sequence reads from FASTQ files were aligned to the mouse genome (GRCm39), downloaded from GENCODE release M27. Alignment was performed using STAR aligner (version2.7.3a). On average, 20 millions of 75 base pairs paired-end reads were generated per sample. The normalized counts of the different genes and isoforms was performed using RSD (version 1.3.1) using a GTF from GENCODE M27 and EnSDbl 104 for gene name annotation. Finally, differential expression was performed using R version 3.6.3 and DESeq2 package v1.24.0. Four biological replicates *per* condition were used.

### Glucose uptake via 2-deoxy-2-[18F] fluoroglucose

Mice were injected with 0.5 MBq/g of ^18^FDG and acquisition of positron emission tomography was assessed. Computed tomography (CT) scans were also acquired for anatomical landmarks and to obtain tissue attenuation coefficients necessary to correct the location of positrons emissions. Standard uptake values (SUV) were calculated using the total weight and the ^18^FDG dose injected to normalize the values. Zones were selected using the Statistical Parametric Map Inveon Research Workplace software (Version 4.2; Siemens Medical Solution).

### Statistical analysis

Results were presented as mean ± SEM. Statistical analyses were performed using 2-Way ANOVA and subsequent Šidák testing for multiple comparison if the treatment condition was significant. Areas under the curve were analysed by unpaired *t*-test, if the *f*-test was statistically significant. In all analyses, *P* < 0.05 was considered statistically significant. *n* refers to the number of animals studied.

## Supplementary Information

Supplementary Information.

## Data Availability

The authors confirm that the data supporting the findings of this study are available within the article and its Supplementary material. Raw data that support the findings of this study are available from the corresponding author, upon reasonable request.
