# Supplementary material for: Chronic NH4Cl loading improves glucose tolerance without modifying insulin sensitivity in mice
Source: Sci Rep. 2026 Feb 3;16:7048. doi: 10.1038/s41598-026-38007-7 (PMC12921001; doi:10.1038/s41598-026-38007-7)
Supplement: Supplementary file 1 — Supplementary Information. [file 41598_2026_38007_MOESM1_ESM.pdf]

Supplementary table 1. **List of antibodies used during protein detection, with their concentrations and the types of samples used.**

Primary antibody	Reference/ supplier	Dilution	Blocking buffer	Sample used	Secondary antibody	Reference/ supplier	Dilution
SGLT2	sc393350/ Santa Cruz	1/100	Milk 1 % in PBS	Kidney Plasma membrane	m-IgG1 BP-HRP	sc525408/ Santa Cruz	1/10000
SGLT1	07-1417/ Merck	1/1600	Milk 1 % in TPBS 0,01%	Kidney Plasma membrane	Goat anti-rabbit IgG HRP	1706515 / Biorad	1/10000
GLUT2	sc518022/ Santa Cruz	1/100	Milk 1 % in PBS	Kidney Plasma membrane	m-IgG1 BP-HRP	sc525408/ Santa Cruz	1/10000
GLUT1	Custom antibody	1/10000	Milk 1 % in PBS	Kidney Plasma membrane	Goat anti-rabbit IgG HRP	1706515 / Biorad	1/10000
PCK1	16754 – 1 – AP/ Proteinintech	1/10000	Milk 1 % in PBS	Kidney lysates	Goat anti-rabbit IgG HRP	1706515 / Biorad	1/10000
		1/50000		Liver lysates			
		1/10000		Intestine homogenates			
G6PC	Kindly provided by Dr Gilles Mithieux and Dr Fabienne Rajas withSupplement an MTA agreement (See reference below)	ary	Milk 1 % in PBS	Kidney lysates	Goat anti-rabbit IgG HRP	1706515 / Biorad	1/10000
		1/50000		Liver lysates			
β-actin	8227/Abcam	1/200000	Milk 1 % in PBS	Kidney Plasma membrane	Goat anti-rabbit IgG HRP	1706515 / Biorad	1/10000
		1/200000		Kidney lysates			
		1/200000		Liver lysates			
		1/200000		Intestine homogenates			

Clar J, Gri B, Calderaro J, Birling MC, Hérault Y, Smit GP, Mithieux G, Rajas F. Targeted deletion of kidney glucose-6 phosphatase leads to nephropathy. Kidney Int. 2014 Oct;86(4):747-56. doi: 10.1038/ki.2014.102. Epub 2014 Apr 9. PMID: 24717294; PMCID: PMC5678048.

Supplementary Table 2. **List of forward and reverse primers used for RNA measurement and profiling**

**Gene**	**Gene ID**	**Forward**	**Reverse**	**Length**	**Tm**	**GC%**	**Ct**
Sglt2	246787	CCCATCCCTCAGAAGCATCTCC	CTCATCCCACAGAACCAAAGCA	22	F-62<br>R-61	F-59<br>R-50	22-25
Sglt1	20537	TCTGTAGTGGCAAGGGGAAG	ACAGGGCTTCTGTGTCTTGG	20	F-59<br>R-60	55	24-25
Glut2	20526	GTTGGAAGAGGAAGTCAGGGCA	ATCACGGAGACCTTCTGCTCAG	22	62	55	23-25
Glut1	20525	GAGTGACGATCTGAGCTACGG	CGTTACTCACCTTGCTGCTG	F-21<br>R-20	F-60<br>R-59	F-57<br>R-55	27-29
Pck1	18534	CCATCCCAACTCGAGATTCTG	CTGAGGGCTTCATAGACAAGG	21	58	52	17-22
G6pc	14377	GGCAAAATGGCAAGGAGACC	CTTGGATGGCTTGGGCTAGG	F-23<br>R-19	60	F-55<br>R-60	21-25
Gapdh	2597	CAAGGTCATCCATGACAACTTTG	GGCCATCCACAGTCTTCTGG	F-23<br>R-20	F-58<br>R-60	F-43<br>R-60	17.52

Supplementary Table 3**. In MA mice' kidney, enrichment of significantly upregulated or downregulated genes in pathways between 3 and 60 days of treatment.** Enrichment analysis was done using Metascape

Days of treatment		Description	InTerm_InList	Gene Symbols
3	Upregulated	Sodium-coupled sulphate, di- and tri-carboxylate transporters	8/-	Slc13a2,Slc13a5,Slc13a4,Slc38a3,Mfsd2a,Slc16a6,Slc51a,Slc25a25
		Metabolism of xenobiotics by cytochrome P450 - Mus musculus (house mouse)	11/-	Cbr2,Gstm1,Hsd11b1,Ugt1a2,Cbr3,Ugt1a1,Rgn,Ces2b,Mat2a,Nr1i3,Fgf18
		allantoin metabolic process	11/-	Nt5c1b,Urah,Dnph1,Noct,Dnase1,Hsd11b1,Nudt19,Ugt1a1,Nccrp1,Cbr2,Slc25a25
		positive regulation of triglyceride biosynthetic process	8/-	Rgn,Plin5,Mfsd2a,Cry1,Fitm1,Pck1,Ugt1a1,Rarres2
		Proximal tubule bicarbonate reclamation - Mus musculus (house mouse)	7/-	Glud1,Pck1,Slc38a3,Eci3,Urah,Cbr3,Nudt19
	Downregulated	lipid transport	20/-	Apoh,Aqp8,Fabp7,Ces1g,Abcb1a,Pla2g5,Ppard,Stra6,Syt7,Stra6l,Ano3,Atp8b4,Slc10a5,Slc6a2,Slc38a4,Slc7a12,Sirpa,Calcrl,Ston1,Stab2
		Eicosanoid metabolism via cyclooxygenases COX	18/-	Cyp4a12b,Pla2g5,Ppard,Tbxas1,Cyp4f14,Cyp1a1,Cyp2d9,Bco1,Lrat,Cyp2d12,Cyp2c69,Scd1,Ces1g,Bhmt,Fos,Gabrb3,Npas2,Ipmk
		isoprenoid transport	5/-	Abcb1a,Stra6,Stra6l,Ano3,Atp8b4
		mechanosensory behavior	8/-	Etv1,Stra6,Slitrk6,Mdk,Fos,Ano3,Npas2,Nr2c2
		PPAR signaling pathway	15/-	Fabp7,Cyp4a12b,Ppard,Scd1,Sorbs1,Apoh,Ces1g,Irs1,Zfp750,Aqp8,Asxl3,Sirpa,Ston1,Vwa2,Mas1
		sodium ion transport	9/-	Cacna1g,Slc6a2,Slc38a4,Slc34a3,Slc10a5,Slc17a4,Gabrb3,Clic6,Ano3

7	Upregulated	anion transport	7/441	BEST1,SLC13A2,SLC38A3,SLC13A4,UGT1A3,SLC26A6,SLC25A25,ABCA1,AQP2,LCN2,FABP5,COX6A2
		uronic acid metabolic process	3/25	DCXR,UGT1A1,UGT1A3,GSTM5,NAT8,RARRES2,PCK1,PTGDS,BACE2,ABCA1
		NR1H2 and NR1H3-mediated signaling	3/47	Atp5b,Atp5c1,Atp5pb,Atp5g1,Atp5j,Atp5k,Cycs,Glud1,Sod2,Atp5l,Atp5o,Atp5j2,Atp5d,Atp5e,Atp5h,Atp5g3,Cdk1,Pde6d,Cetn2,Ppp2r1a,Nphp1, Dynlrb1,Ift172,Sdccag8
		Glutathione metabolism	3/57	ABCA1,PCK1,UGT1A3,FABP5,H3C3,MOGAT2
		digestion	3/108	CCK,SLC26A6,MOGAT2
		glucose metabolic process	3/113	FABP5,PCK1,DCXR,ABCA1,PTGDS,MOGAT2,FITM1,PHOSPHO1,BACE2
	Downregulated	regulation of secretion	7/771	Ces1g,Cyp27b1,Irs1,Septin2,Syt7,Exph5,Zbed6,Prr5l
		cerebellum development	3/111	Atp7a,Mdk,Nr2c2
		regulation of generation of precursor metabolites and energy	3/149	Atp7a,Irs1,Sorbs1,Ces1g,Cyp27b1
		organic hydroxy compound transport	3/160	Ces1g,Syt7,Slc10a5,Spns3
		organic hydroxy compound biosynthetic process	3/171	Atp7a,Ces1g,Cyp27b1

14	Upregulated	Proximal tubule bicarbonate reclamation - Mus musculus (house mouse)	4/22	Acadm,Bcat1,Cdo1,Gls,Glud1,Hdc,Phyh,Nos3,Pck1,Echdc2,Hibadh,Eci3,Afmid,Crot,Acox3,Aldh1l1,Shmt2,Akr1d1,Csad,Ahcy,Amt,Apobec2,Akr1c18,Kyat1,Dglucy,Tyms,Dmgdh,Gpx6,Suox
		skeletal system development	7/536	Ace,Slc10a2,Slc13a2,Slc23a1,Ceacam2,Mpc1,Slc16a9,Ace2,Slc38a3,Slc6a20a,Slc6a15,Slc16a6,Slc26a6,Slc22a19,Slc5a8,Slco4c1,Slc16a4,Slc6a17,Slc35a3,Slc22a4,Crot,Slc9a2
		Adipocytokine signaling pathway - Mus musculus (house mouse)	3/71	Ace,Angpt2,Cfh,Enpep,Fn1,Anpep,Lepr,Nos3,Notch3,Notch4,Nrp2,Pdgfra,Pitx2,Cxcl12,Tcf21,Grem1,Clec14a,Plxnd1,Pxdn,Robo4,Celsr1,Fgfr3,Tnc,Wnt9b,Wnt5a,Ift122,Alx1,Dchs1,Kif20b,Stard13,Col4a3,Nid1,Nup107,Cat,Clcnka,Fbn1,Nsdhl
		Metabolism of xenobiotics by cytochrome P450 - Mus musculus (house mouse)	3/73	Bst1,Gpc1,Nos3,Slc23a1,Tpk1,Parp8,Akr1c18,Akr1c14,Aldh1l1,Shmt2,Bco2,Pdxk,Slc5a8,Gphn
		organic hydroxy compound metabolic process	6/469	Slc13a2,Slc14a2,Slc22a4,Slc38a3,Slc6a20a,Slc6a15,Slc12a6,Slc14a1,Slc26a6,Slc5a8,Slc9a2,Slco4c1,Slc35a3,Slc13a4,Aco1,Aqp3,Car2,Clcn2,Clcnka,Pcsk6,Abcc9,Zdhhc8,Atp2a3,Slc22a19
	Downregulated	plasma lipoprotein particle organization	4/38	Apoc1,Ces1g,Pla2g5,Apom,Fabp1,Stra6,Nme4,Spns3,Atm
		Complement and coagulation cascades - Mus musculus (house mouse)	5/93	C2,Cfi,Kng1,Tfpi,Fgb,Mdk,Apoc1,Fabp1,Renbp
		digestion	4/92	Fabp1,Mdk,Snx10,Adm2
		amino sugar metabolic process	3/45	Renbp,Npl,Uap1l1,Cyp27b1,Acaa2,Acmsd

		acute inflammatory response	3/70	Hp,Orm1,Mylk3,Kng1,Mdk,Cela1
30	Upregulated	Proximal tubule bicarbonate reclamation - Mus musculus (house mouse)	4/22	Gls,Glud1,Pck1,Slc38a3
		intestinal absorption	3/29	Rsc1a1,Slc26a6,Mogat2
		Metabolism of xenobiotics by cytochrome P450 - Mus musculus (house mouse)	3/73	Cbr2,Ugt2b5,Ugt2b37
		TGF-beta signaling pathway - Mus musculus (house mouse)	3/95	Fst,Grem1,Grem2,Gpc1,Nat8f5
		carboxylic acid transport	4/229	Slc13a2,Slc38a3,Slc16a6,Slc26a6
	Downregulated	cellular amino acid metabolic process	22/250	Ass1,Dao,Ddc,Dio1,Dpep1,Glul,Oat,Prodh,Aadat,Plod2,Gcat,Cars,Prodh2,Thap4,Slc39a8,Amdhd1,Agmat,Psat1,Asl,Gm4952,Uroc1,Acmsd,Amd1,Crym,Rida,Slc25a15,Folh1,Gatm,Tstd1,Folr1,Qprt,Ogdhl,Apoe,Cyp27b1,Renbp,Acaa2,Miox,Oxct1,Dhdh,Npl,Gatd1,Amdhd2
		organic hydroxy compound metabolic process	23/469	Apoe,Ces1g,Comt,Crym,Cyp2d9,Cyp27b1,Dao,Ddc,Dio1,Ces1e,Gc,Ly6e,Mttp,Dhrs3,Hao2,Miox,Ebpl,Ipmk,Degs2,Cyp2d26,Gatd1,Rdh16f2,Cyp2d12,Hsd3b4,Hint2,Hsd3b9,Acsl1,Fmo5,Gstt1,Vkorc1,As3mt,Cyp2c69,Cpe,Dpep1,Hsd3b8,Acmsd,Gabrb3,Glul,Nucb2,Cltrn,Camk2n1,Oxct1,Fgb,Pla2g5,Tbxas1,Acot1,Hint1,Rida,Qprt,Trir,Nt5c3,Dna2,Pla2g4b
		arginine metabolic process	5/15	Ass1,Oat,Slc39a8,Agmat,Asl,Ces1g,Cyp27b1,Degs1,Ces1e,Fbp2,Glul,Pla2g5,Tbxas1,Plod2,Vkorc1,Gatm,Acss1,Ipmk,Psat1,Gulo,Slc25a15,Ddc,Prodh,Folh1
		Complement and coagulation	9/93	C2,C4b,Cfi,F2,Cfb,Proc,Serpina1a,Tfpi,Fgb,Pla2g5,Cpn1,Sirpa

		cascades - Mus musculus		
60	Upregulated	Proximal tubule bicarbonate reclamation - Mus musculus (house mouse)	4/22	Gls,Glud1,Pck1,Slc38a3,Arg2,Atp7a
		cellular response to pH	3/26	Hyal1,Pck1,Slc38a3,Alb,Wnt2b,Kif26a,Slc25a25
		anion transport	8/406	Slc34a2,Slc16a14,Slc38a3,Slc16a6,Slc26a7,Slc26a10,Slc4a7,Slc10a5
		maintenance of location in cell	4/107	Alb,Syne1,Dzip1,Ccdc88a
		regulation of glucose metabolic process	4/114	Irs1,Sorbs1,Dgkq,Pdk1
	Downregulated	Complement cascade	4/46	C2,Cfi,F2,Masp2,Igkc
		Cytochrome P450 - arranged by substrate type	4/65	Cyp24a1,Cyp27b1,Cyp4b1,Tbxas1,Inmt,Miox,Slc34a3
		organic hydroxy compound metabolic process	8/469	Ces1g,Cyp24a1,Cyp27b1,Ly6e,Miox,Ebpl,Degs2,Cyp2d12
		organic hydroxy compound transport	4/160	Ces1g,Ly6e,Stra6,Apom,Nme4,Spns3

		monocarboxylic acid metabolic process	7/544	Ces1g,Cyp24a1,Tbxas1,Phgdh,Cyp2d12,Ugt1a6b,Cyp2c69,Cyp27b1,Gabrb3

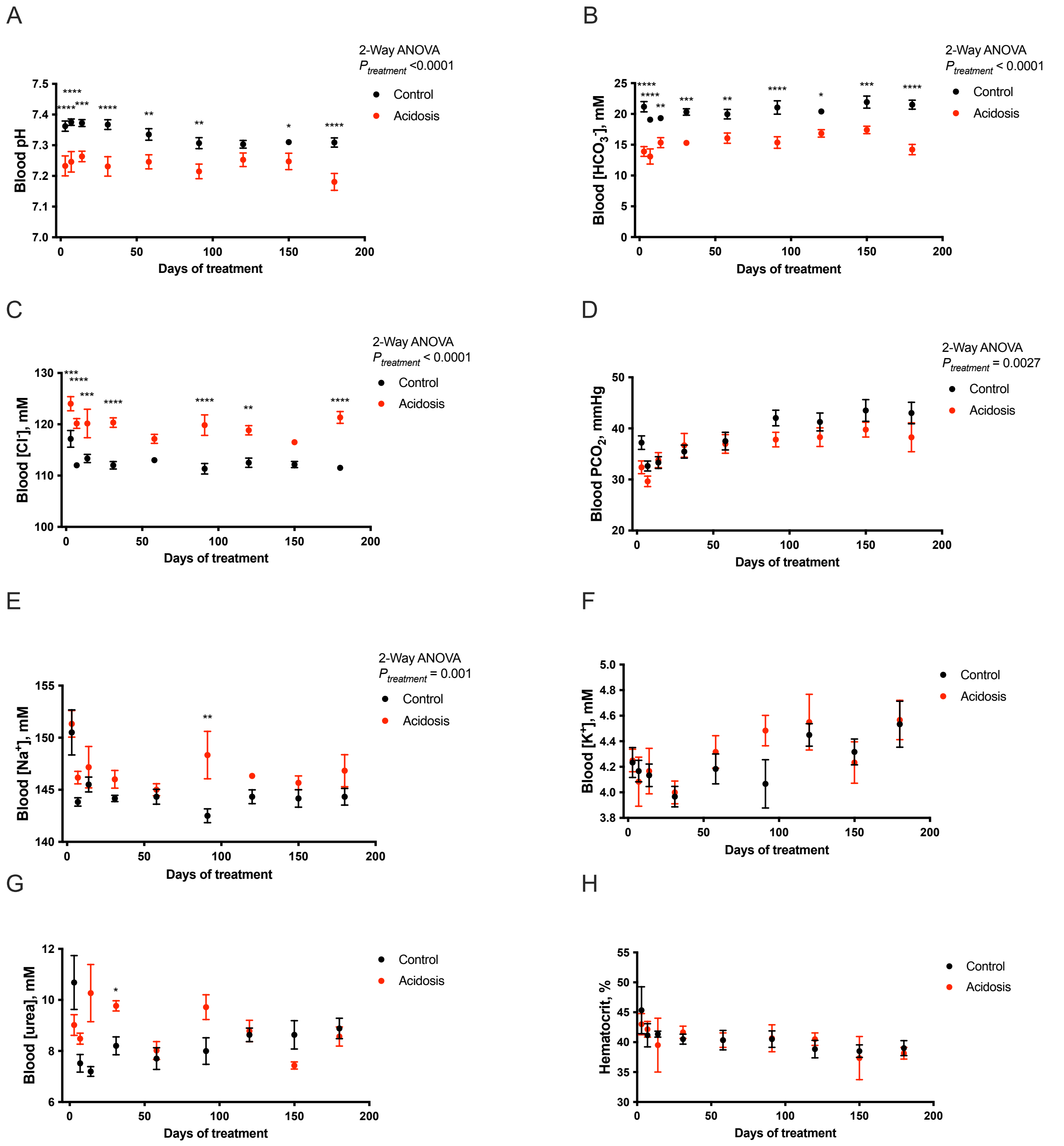

Supplementary figure 1. **Blood parameters of mice confirm metabolic acidosis in treated mice. (A-H)** Blood pH **(A),** HCO_3 concentration **(B),** Cl^- concentration **(C),** PCO_2 **(D),** Na^{2+} concentration **(E),** K^+ concentration **(F),** urea concentration **(G)** and hematocrit percentage **(H)** measured by i-STAT EC8+ cartridge and an i-STAT1 handheld analyser with fresh retro-orbital blood samples between 3 and 180 days of NH_4Cl treatment in control (black) and acidotic (red) mice. All values are expressed as mean ± SEM. Statistical analyses were done using 2-Way ANOVA and subsequent Šidák testing for multiple comparison if the treatment condition was significant, *n* = 6 per group, *$P < 0.05$. **$P < 0.01$, ***$P < 0.001$, ****$P < 0.0001$.

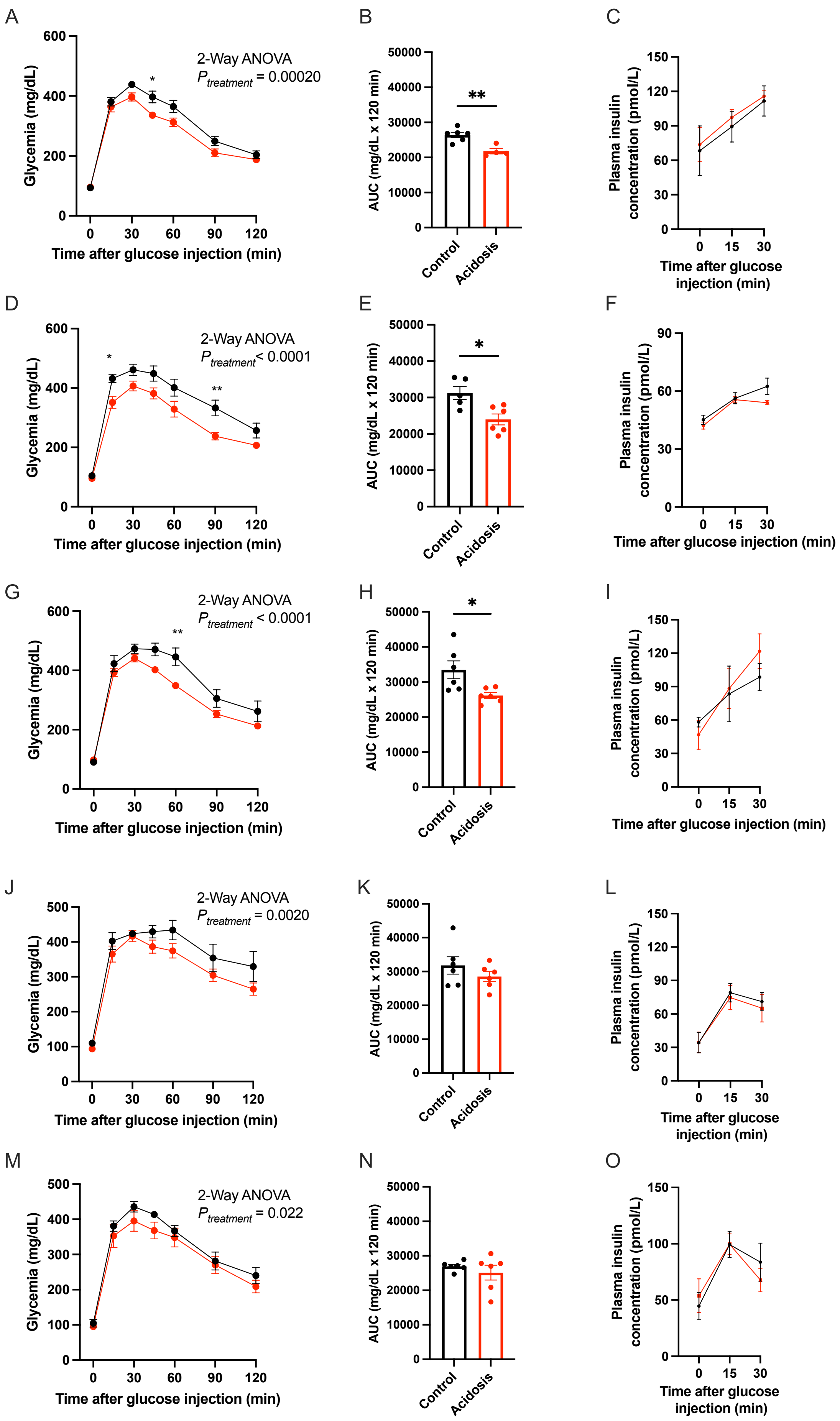

A
2-Way ANOVA
$P_{treatment}$ = 0.00020
Glycemia (mg/dL)
Time after glucose injection (min)
B
AUC (mg/dL x 120 min)
Control
Acidosis
C
Plasma insulin concentration (pmol/L)
Time after glucose injection (min)
D
2-Way ANOVA
$P_{treatment}$ < 0.0001
Glycemia (mg/dL)
Time after glucose injection (min)
E
AUC (mg/dL x 120 min)
Control
Acidosis
F
Plasma insulin concentration (pmol/L)
Time after glucose injection (min)
G
2-Way ANOVA
$P_{treatment}$ < 0.0001
Glycemia (mg/dL)
Time after glucose injection (min)
H
AUC (mg/dL x 120 min)
Control
Acidosis
I
Plasma insulin concentration (pmol/L)
Time after glucose injection (min)
J
2-Way ANOVA
$P_{treatment}$ = 0.0020
Glycemia (mg/dL)
Time after glucose injection (min)
K
AUC (mg/dL x 120 min)
Control
Acidosis
L
Plasma insulin concentration (pmol/L)
Time after glucose injection (min)
M
2-Way ANOVA
$P_{treatment}$ = 0.022
Glycemia (mg/dL)
Time after glucose injection (min)
N
AUC (mg/dL x 120 min)
Control
Acidosis
O
Plasma insulin concentration (pmol/L)
Time after glucose injection (min)

Supplementary figure 2. **Glucose tolerance is ameliorated in chronic metabolic acidosis, without changes to insulin secretion.** **(A-C)** intraperitoneal glucose tolerance test (ipGTT) **(A)** in control (black) and acidotic (red) mice after 15 days of NH_4Cl treatment (*n* = 6 for the control and 4 for the acidotic group) with the corresponding area under the curve (AUC) **(B)** and plasmatic insulin concentration of ipGTT **(C)**. **(D-F)** ipGTT **(D)** in control and acidotic mice after 60 days of NH_4Cl treatment (*n* = 5 for the control and 6 for the acidotic group) with the corresponding AUC **(E)** and plasmatic insulin concentration of ipGTT **(F)**. **(G-I)** ipGTT **(G)** in control and acidotic mice under chow diet after 90 days of NH_4Cl treatment (*n* = 6 per group) with the corresponding AUC **(H)** and plasmatic insulin concentration of ipGTT **(I)**. **(J-L)** ipGTT **(J)** in control and acidotic mice under chow diet after 150 days of NH_4Cl treatment (*n* = 6 per group) with the corresponding AUC **(K)** and plasmatic insulin concentration of ipGTT **(L)**. **(M-O)** ipGTT **(M)** in control and acidotic mice under chow diet after 180 days of NH_4Cl treatment (*n* = 6 per group) with the corresponding AUC **(N)** and plasmatic insulin concentration of ipGTT **(O)**. All values are expressed as mean ± SEM. Statistical analyses were done using 2-Way ANOVA and subsequent Šidák testing for multiple comparison if the treatment condition was significant. Areas under the curve and basal plasmatic insulin concentration were analysed by unpaired t-test, if the f-test is statistically significant, then a Welsh's *t*-test was done. **P* < 0.05. ***P* < 0.01. ns: not significant.

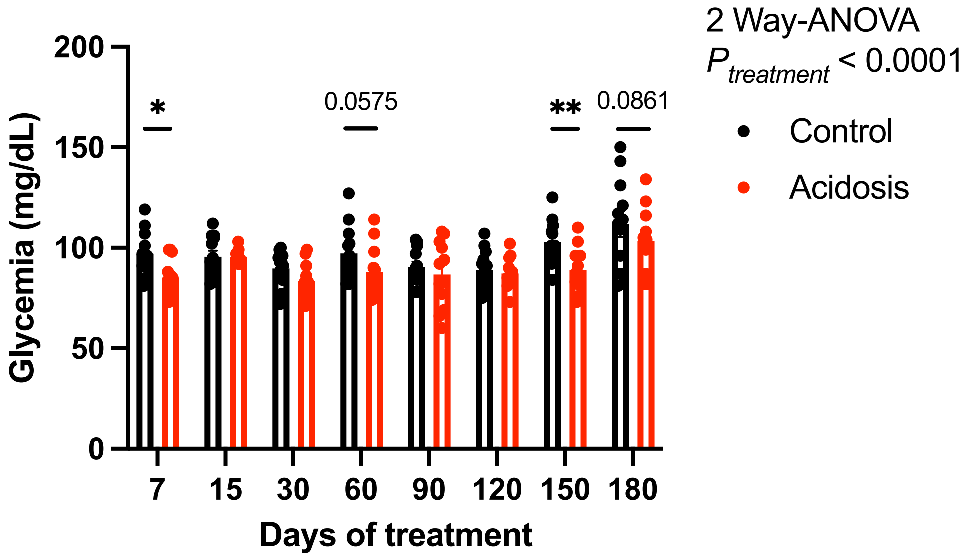

Supplementary figure 3. **Fasting glycemia is significantly lower in MA mice compared to their control.** Blood glucose levels measured using the Accu-Check Performa glucometer after 7, 15, 30, 60, 90, 120, 150 and 180 days of treatment (*n* = 12 per group). Glycemias were taken after a 15 hour fast and before intraperitoneal injection. All values are expressed as mean ± SEM. Statistical analyses were done using 2-Way ANOVA and subsequent Šidák testing for multiple comparison if the treatment condition was significant. $*P < 0.05$. $**P < 0.01$.

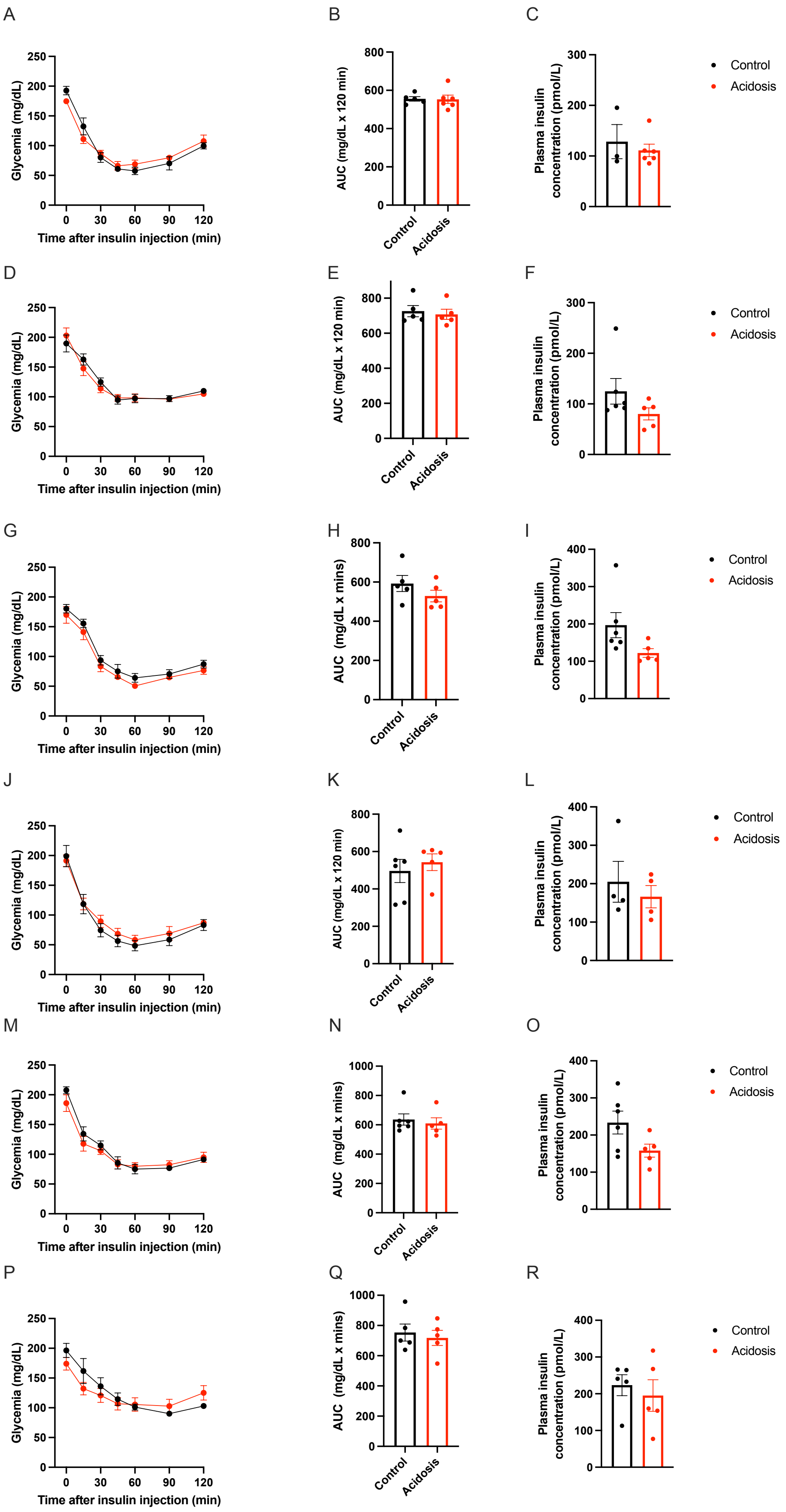

A
Glycemia (mg/dL)
Time after insulin injection (min)
B
AUC (mg/dL x 120 min)
Control
Acidosis
C
Plasma insulin concentration (pmol/L)
Control
Acidosis
D
Glycemia (mg/dL)
Time after insulin injection (min)
E
AUC (mg/dL x 120 min)
Control
Acidosis
F
Plasma insulin concentration (pmol/L)
Control
Acidosis
G
Glycemia (mg/dL)
Time after insulin injection (min)
H
AUC (mg/dL x mins)
Control
Acidosis
I
Plasma insulin concentration (pmol/L)
Control
Acidosis
J
Glycemia (mg/dL)
Time after insulin injection (min)
K
AUC (mg/dL x 120 min)
Control
Acidosis
L
Plasma insulin concentration (pmol/L)
Control
Acidosis
M
Glycemia (mg/dL)
Time after insulin injection (min)
N
AUC (mg/dL x mins)
Control
Acidosis
O
Plasma insulin concentration (pmol/L)
Control
Acidosis
P
Glycemia (mg/dL)
Time after insulin injection (min)
Q
AUC (mg/dL x mins)
Control
Acidosis
R
Plasma insulin concentration (pmol/L)
Control
Acidosis

Supplementary figure 4. **Insulin sensitivity does not differ during chronic metabolic acidosis. (A-C)** intraperitoneal insulin tolerance test (ipITT) **(A)** in control (black) and acidotic (red) mice after 15 days of NH_4Cl treatment (n = 5 for the control and 6 for the acidotic group) with the corresponding area under the curve (AUC) **(B)** and basal plasmatic insulin concentration **(C)** of ipITT. **(D-F)** ipITT **(D)** in control and acidotic mice after 30 days of NH_4Cl treatment (n = 5 per group) with the corresponding AUC **(E)** and basal plasmatic insulin concentration **(F)** of ipITT. **(G-I)** ipITT **(G)** in control and acidotic mice after 60 days of NH_4Cl treatment (n = 5 per group) with the corresponding AUC **(H)** and basal plasmatic insulin concentration **(I)** of ipITT. **(J-L)** ipITT **(J)** in control and acidotic mice after 90 days of NH_4Cl treatment (n = 6 for the control and 5 for the acidotic group) with the corresponding AUC **(K)** and basal plasmatic insulin concentration **(L)** of ipITT. **(M-O)** ipITT **(M)** in control and acidotic mice after 150 days of NH_4Cl treatment (n = 6 for the control and 5 for the acidotic group) with the corresponding AUC **(N)** and basal plasmatic insulin concentration **(O)** of ipITT. **(P-R)** ipITT **(P)** in control and acidotic mice under chow diet after 180 days of NH_4Cl treatment (n = 5 per group) with the corresponding AUC **(Q)** and basal plasmatic insulin concentration **(R)** of ipITT. All values are expressed as mean ± SEM. Statistical analyses were done using 2-Way ANOVA and subsequent Šidák testing for multiple comparison if the treatment condition was significant. Areas under the curve and basal plasmatic insulin concentration were analysed by unpaired *t*-test, if the *f*-test is statistically significant, then a Welsh's *t*-test was done.

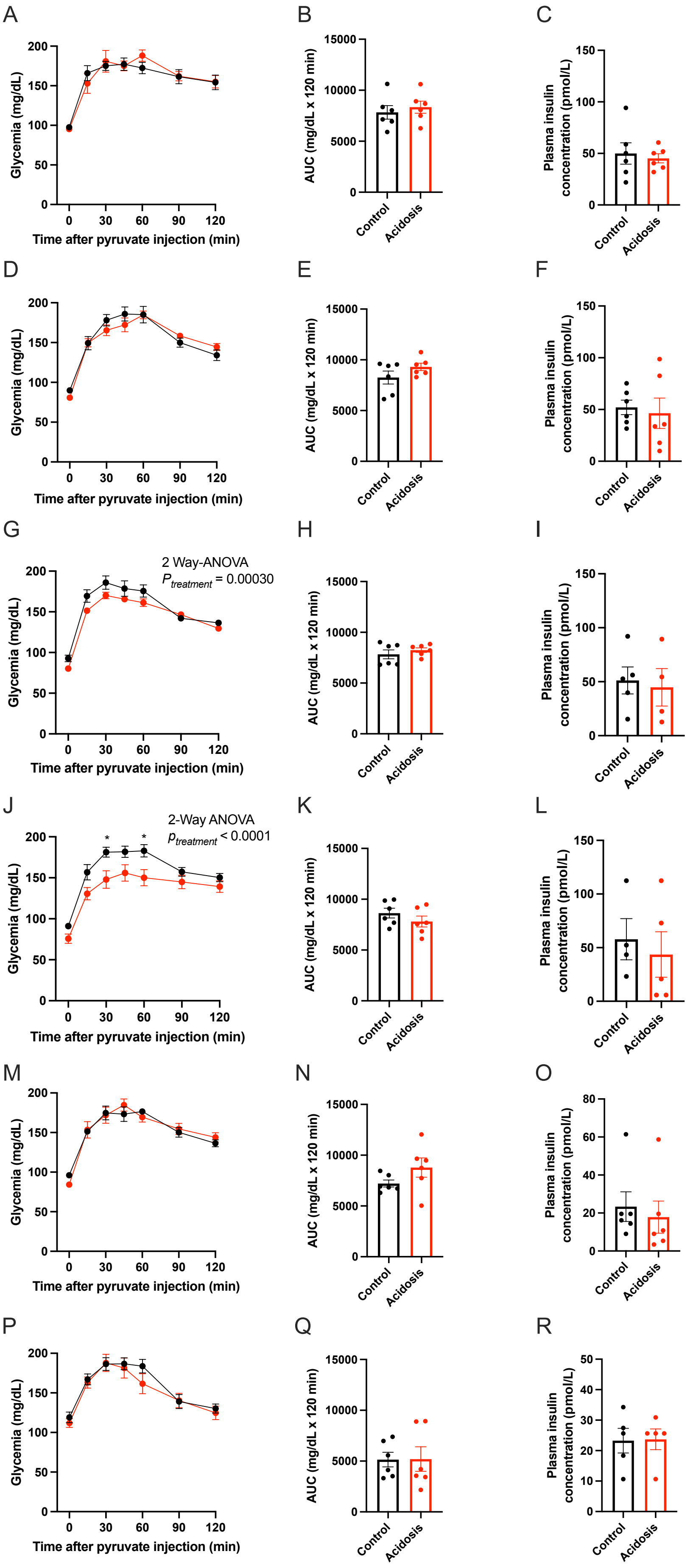

A
Glycemia (mg/dL)
Time after pyruvate injection (min)
B
AUC (mg/dL x 120 min)
Control
Acidosis
C
Plasma insulin concentration (pmol/L)
D
E
F
G
2 Way-ANOVA
$P_{treatment}$ = 0.00030
H
I
J
2-Way ANOVA
$p_{treatment}$ < 0.0001
K
L
M
N
O
P
Q
R

Supplementary figure 5. **Total gluconeogenesis is altered during chronic metabolic acidosis.** **(A-C)** Intraperitoneal pyruvate tolerance test (ipITT), **(A)** in control (black) and acidotic (red) mice after 15 days of NH_4Cl treatment (*n* = 6 per group) with the corresponding area under the curve (AUC) **(B)** and basal plasmatic insulin concentration **(C)** of ipPTT. **(D-F)** ipPTT **(D)** in control and acidotic mice after 30 days of NH_4Cl treatment (*n* = 6 per group) with the corresponding AUC **(E)** and basal plasmatic insulin concentration **(F)** of ipPTT. **(G-I)** ipPTT **(G)** in control and acidotic mice after 60 days of NH_4Cl treatment (*n* = 6 per group) with the corresponding AUC **(H)** and basal plasmatic insulin concentration **(I)** of ipPTT (*n* = 5 for the control and 4 for the acidotic group). **(J-L)** ipPTT (J) in control and acidotic mice after 90 days of NH_4Cl treatment (*n* = 6 per group) with the corresponding AUC **(K)** and basal plasmatic insulin concentration **(L)** of ipPTT (*n* = 4 for the control and 5 for the acidotic group). **(M-O)** ipPTT **(M)** in control and acidotic mice after 150 days of NH_4Cl treatment (*n* = 6 per group) with the corresponding AUC **(N)** and basal plasmatic insulin concentration **(O)** of ipPTT. **(P-R)** ipPTT **(P)** in control and acidotic mice under chow diet after 180 days of NH_4Cl treatment (*n* = 6 per group) with the corresponding AUC **(Q)** and basal plasmatic insulin concentration **(R)** of ipPTT (*n* = 5 per group). All values are expressed as mean ± SEM. Statistical analyses were done using 2-Way ANOVA and subsequent Šidák testing for multiple comparison if the treatment condition was significant. Areas under the curve and basal plasmatic insulin concentration were analysed by unpaired *t*-test, if the *f*-test is statistically significant then a Welsh's *t*-test was done. $*P < 0.05$.

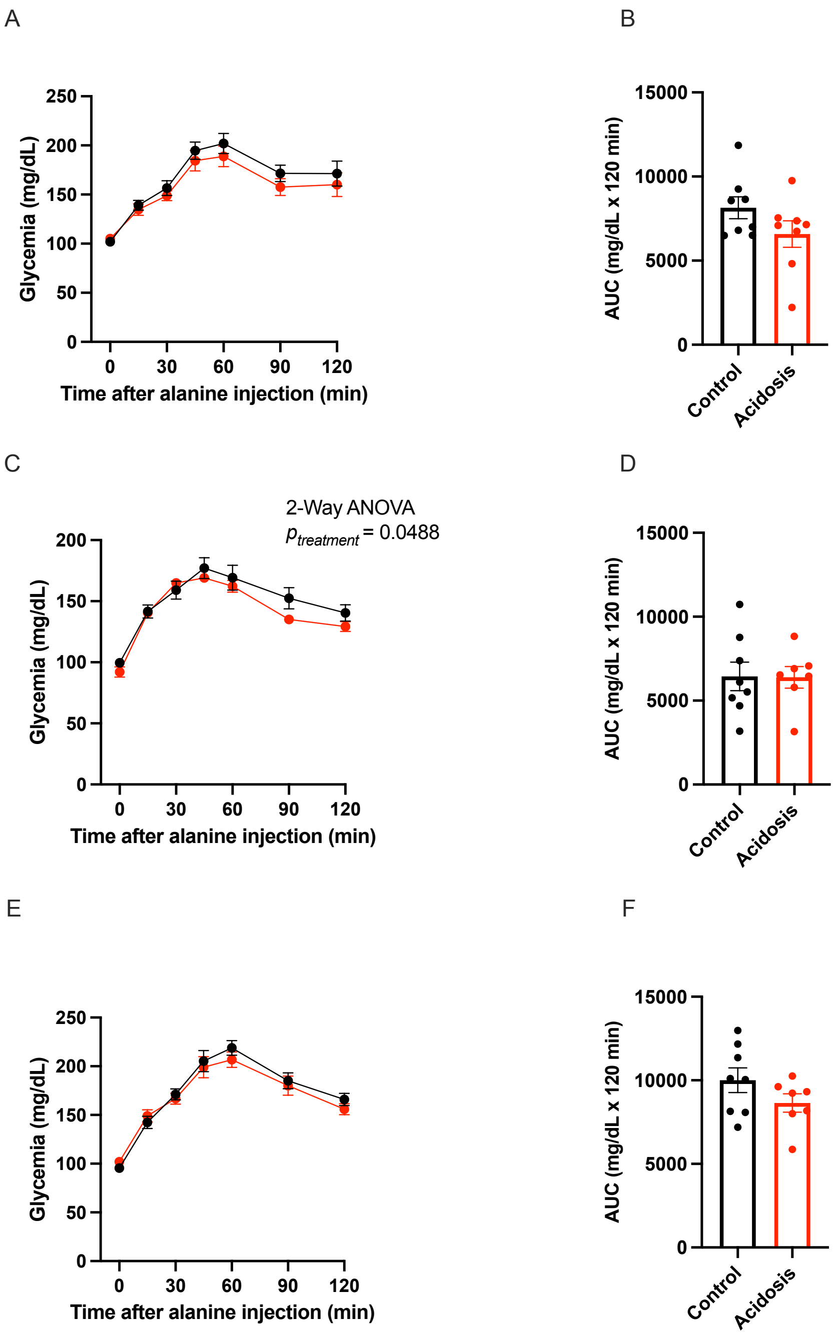

Supplementary figure 6. **Hepatic endogenous glucose production is diminished during chronic MA. (A, B)** Intraperitoneal alanine tolerance test (ipAlaTT) **(A)** in control (black) and acidotic (red) mice under chow diet after 30 days of NH_4Cl treatment (n = 8 per group) with the corresponding area under the curve (AUC) **(B)** of ipAlaTT. **(C, D)** ipAlaTT **(C)** in control and acidotic mice under chow diet after 60 days of NH_4Cl treatment (n = 8 for the control and 7 for the acidotic group) with the AUC **(D)** of ipAlaTT. **(E, F)** ipAlaTT **(E)** in control and acidotic mice under chow diet after 90 days of NH_4Cl treatment (n = 8 for the control and 7 for the acidotic group) with the AUC **(F)** of ipAlaTT. All values are expressed as mean ± SEM. Statistical analyses were done using 2-Way ANOVA and subsequent Šidák testing for multiple comparison if the treatment condition was significant. Areas under the curve were analysed by unpaired t-test, if the f-test is statistically significant then a Welsh's t-test was done. $^*P < 0.05$.

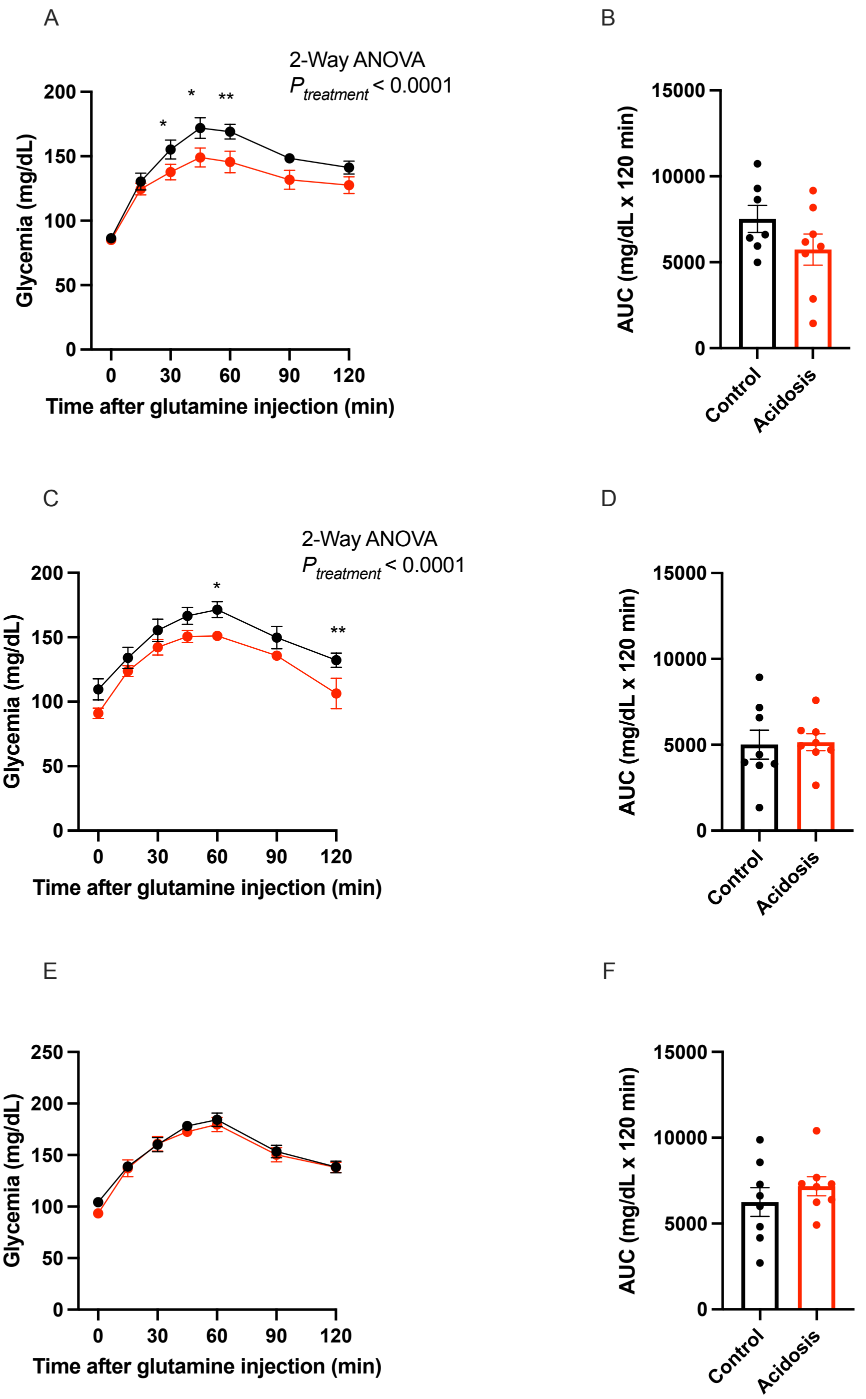

Supplementary figure 7. **Renal/intestinal endogenous glucose production is diminished during chronic MA. (A, B)** Intraperitoneal glutamine tolerance test (ipGluTT) **(A)** in control (black) and acidotic (red) mice under chow diet after 30 days of NH_4Cl treatment ($n = 7$ for the control and 8 for the acidotic group) with the corresponding area under the curve (AUC) **(B)** of ipGluTT. **(C, D)** ipGluTT **(C)** in control and acidotic mice under chow diet after 60 days of NH_4Cl treatment ($n = 8$ per group) with the AUC **(D)** of ipGluTT. **(E, F)** ipGluTT **(E)** in control and acidotic mice under chow diet after 90 days of NH_4Cl treatment ($n = 8$ per group) with the AUC **(F)** of ipGluTT. All values are expressed as mean ± SEM. Statistical analyses were done using 2-Way ANOVA and subsequent Šidák testing for multiple comparison if the treatment condition was significant. Areas under the curve were analysed by unpaired *t*-test, if the *f*-test is statistically significant then a Welsh's *t*-test was done. $^*P < 0.05$, $^{**}P < 0.01$.

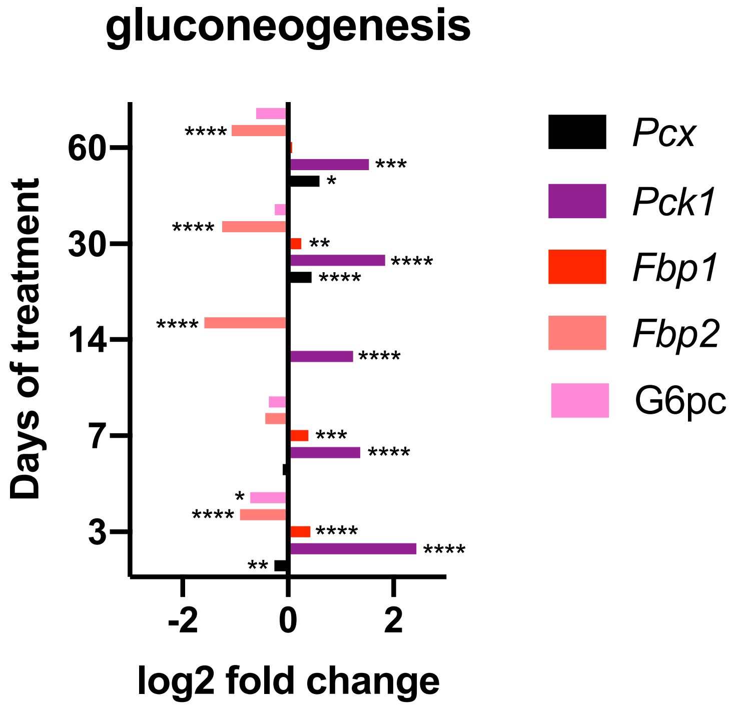

Supplementary figure 8. **Renal gluconeogenesis is altered by chronic acidosis during 3 and 60 days of treatment.** Log2 fold change of mRNA expression of *Pcx, Pck1, Fbp1, Fbp2* and *G6pc* in control and acidotic mice' kidney between 3 and 60 days of treatment analysed by RNA sequencing ($n = 4$ per group). All values are expressed as Log2 fold change values; $*P < 0.05$, $**P < 0.01$, $***P < 0.001$, $****P < 0.0001$.

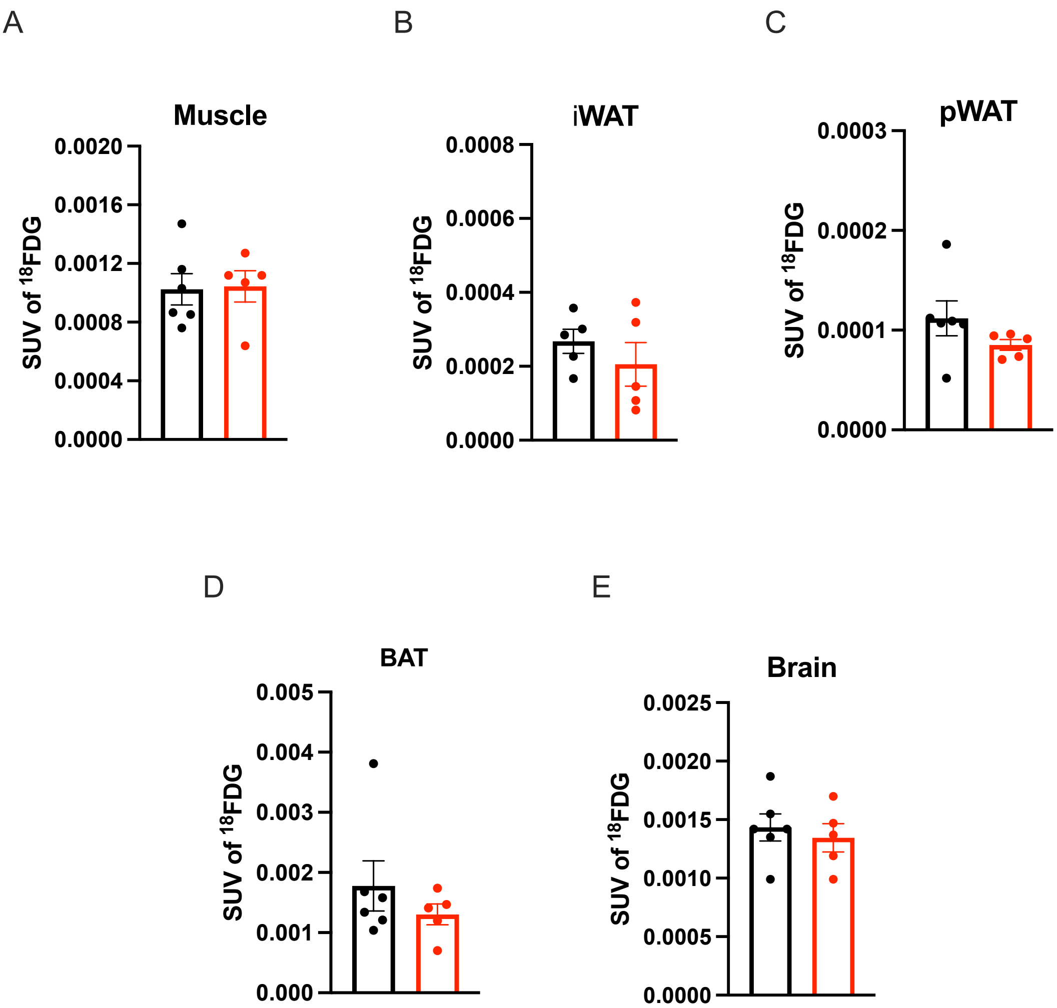

Supplementary figure 9. **180 days of NH_4Cl treatment does not alter whole body 2-Deoxy-2-[18F]fluoroglucose uptake. (A-E)** Standard uptake values (SUV) of 2-Deoxy-2-[18F]fluoroglucose (^{18}FDG) in the skeletal muscle **(A),** inguinal white adipose tissue **(B)**, perigonadal white adipose tissue **(C),** brown adipose tissue **(D)** and whole brain **(E)** of control (black) and acidotic (red) mice after 180 days of NH_4Cl treatment (n = 6 for the control and 5 for the acidotic mice). SUV's were analysed by unpaired *t*-test, if the *f*-test is statistically significant then a Welsh's *t*-test was done. All values are expressed as mean ± SEM. SUV: standard uptake value.

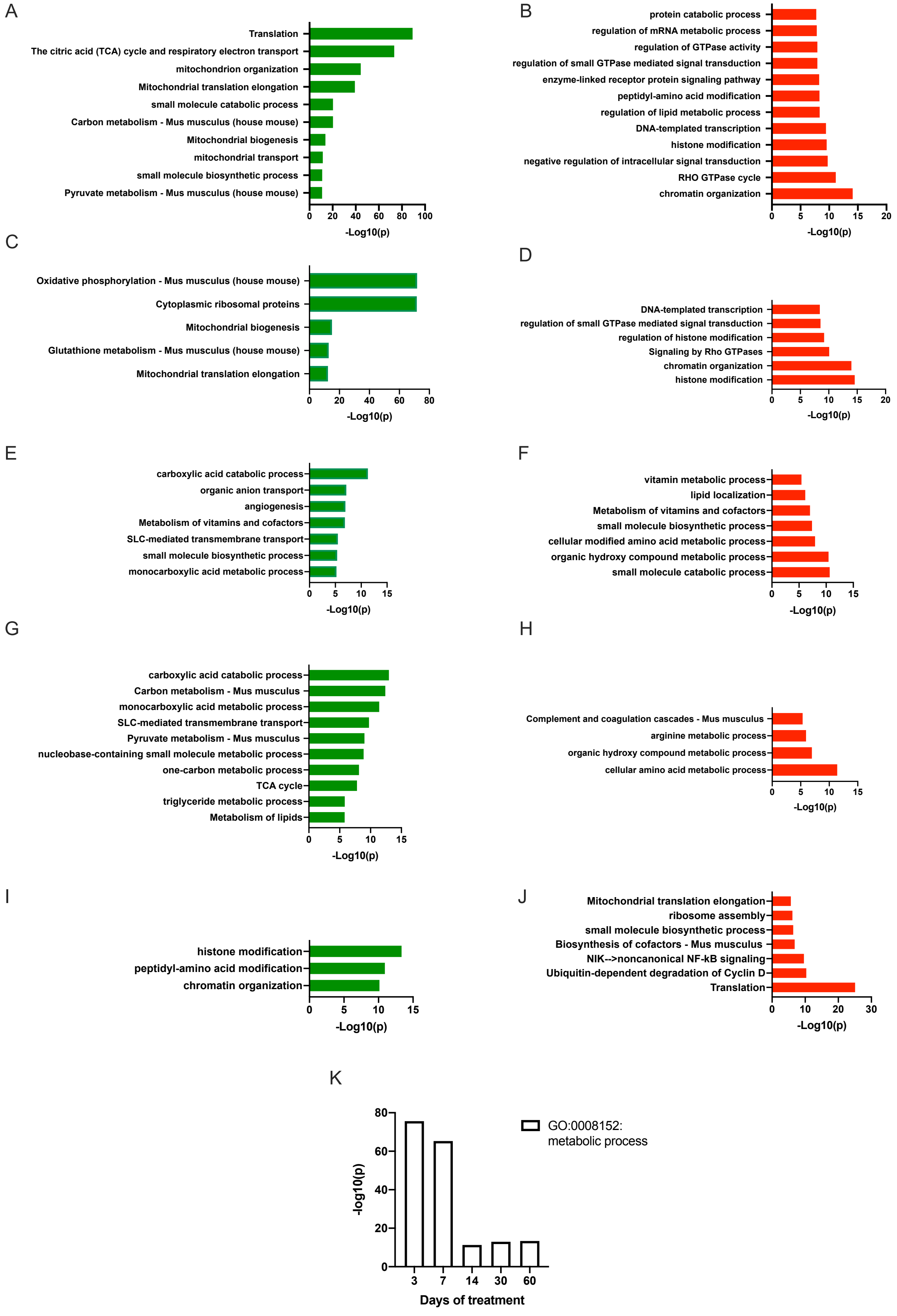
A
Translation
The citric acid (TCA) cycle and respiratory electron transport
mitochondrion organization
Mitochondrial translation elongation
small molecule catabolic process
Carbon metabolism - Mus musculus (house mouse)
Mitochondrial biogenesis
mitochondrial transport
small molecule biosynthetic process
Pyruvate metabolism - Mus musculus (house mouse)
-Log10(p)
B
protein catabolic process
regulation of mRNA metabolic process
regulation of GTPase activity
regulation of small GTPase mediated signal transduction
enzyme-linked receptor protein signaling pathway
peptidyl-amino acid modification
regulation of lipid metabolic process
DNA-templated transcription
histone modification
negative regulation of intracellular signal transduction
RHO GTPase cycle
chromatin organization
-Log10(p)
C
Oxidative phosphorylation - Mus musculus (house mouse)
Cytoplasmic ribosomal proteins
Mitochondrial biogenesis
Glutathione metabolism - Mus musculus (house mouse)
Mitochondrial translation elongation
-Log10(p)
D
DNA-templated transcription
regulation of small GTPase mediated signal transduction
regulation of histone modification
Signaling by Rho GTPases
chromatin organization
histone modification
-Log10(p)
E
carboxylic acid catabolic process
organic anion transport
angiogenesis
Metabolism of vitamins and cofactors
SLC-mediated transmembrane transport
small molecule biosynthetic process
monocarboxylic acid metabolic process
-Log10(p)
F
vitamin metabolic process
lipid localization
Metabolism of vitamins and cofactors
small molecule biosynthetic process
cellular modified amino acid metabolic process
organic hydroxy compound metabolic process
small molecule catabolic process
-Log10(p)
G
carboxylic acid catabolic process
Carbon metabolism - Mus musculus
monocarboxylic acid metabolic process
SLC-mediated transmembrane transport
Pyruvate metabolism - Mus musculus
nucleobase-containing small molecule metabolic process
one-carbon metabolic process
TCA cycle
triglyceride metabolic process
Metabolism of lipids
-Log10(p)
H
Complement and coagulation cascades - Mus musculus
arginine metabolic process
organic hydroxy compound metabolic process
cellular amino acid metabolic process
-Log10(p)
I
histone modification
peptidyl-amino acid modification
chromatin organization
-Log10(p)
J
Mitochondrial translation elongation
ribosome assembly
small molecule biosynthetic process
Biosynthesis of cofactors - Mus musculus
NIK-->noncanonical NF-kB signaling
Ubiquitin-dependent degradation of Cyclin D
Translation
-Log10(p)
K
-log10(p)
GO:0008152:
metabolic process
3
7
14
30
60
Days of treatment

Supplementary figure 10. **Mitochondrial activity, oxidative metabolism and catabolic pathways are upregulated in the kidney during chronic metabolic acidosis.** Functional enrichment results in acidotic mice compared to their control after 3 (A-B), 7 (C-D), 14 (E-F), 30 (G-H) and 60 (I-J) days of NH_4Cl treatment. Summary pathways analysed by Metascape with the highest -log10(p) values were selected. (K) Metabolic process Gene Ontology (GO) membership parent data of acidotic mice vs control expressed in -log10(p) between 3 and 60 days of treatment. *n* = 4 per group.

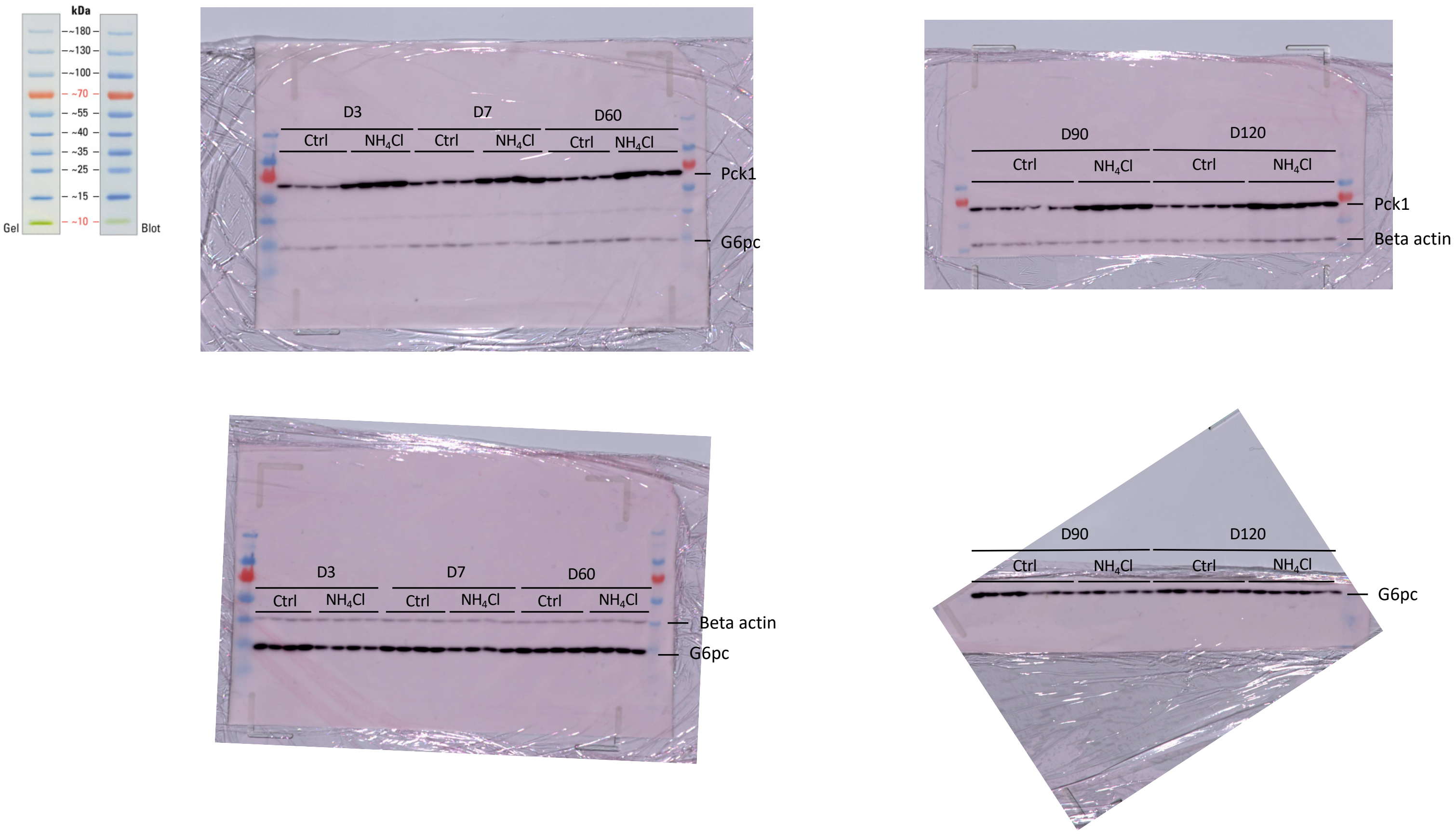

Supplementary Figure 11. **Full length western blots of Figure 4.C**

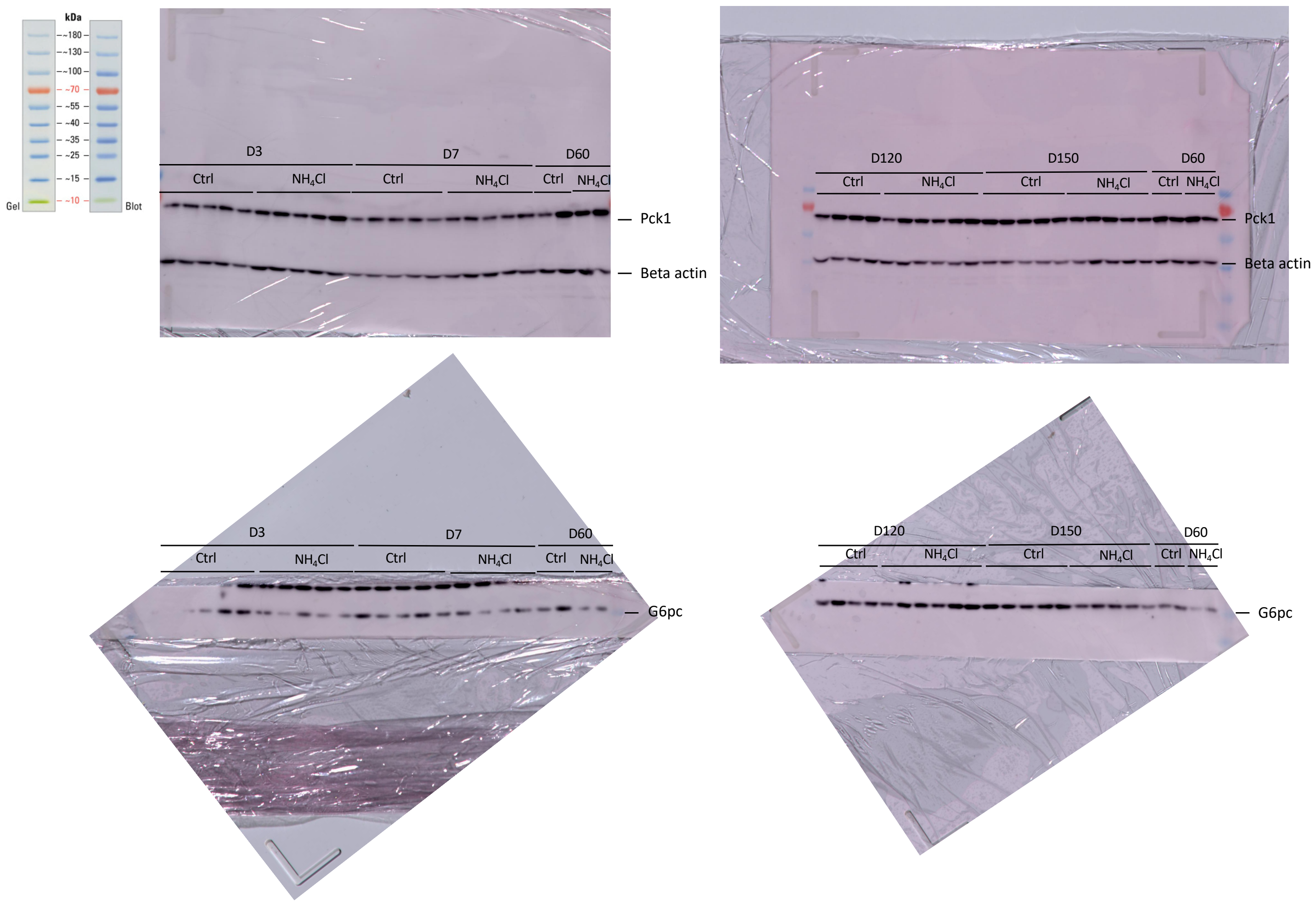

Supplementary Figure 12. **Full length western blots of Figure 4.G**

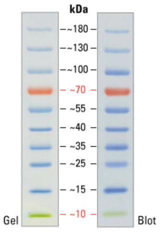
kDa
~180
~130
~100
~70
~55
~40
~35
~25
~15
~10
Gel
Blot

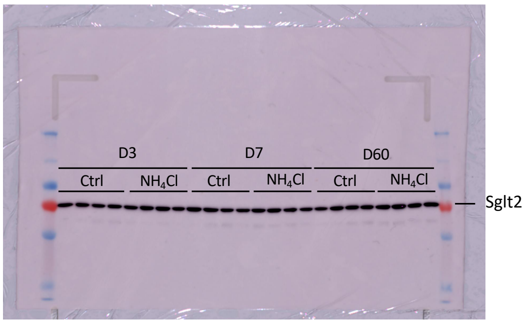
D3
D7
D60
Ctrl
NH₄Cl
Ctrl
NH₄Cl
Ctrl
NH₄Cl
Sglt2

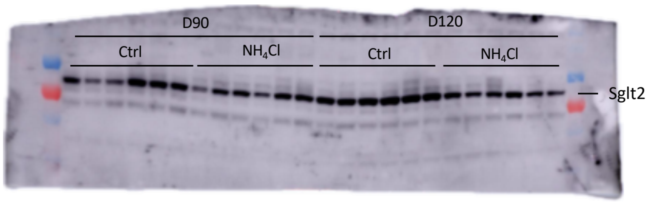
D90
D120
Ctrl
NH₄Cl
Ctrl
NH₄Cl
Sglt2

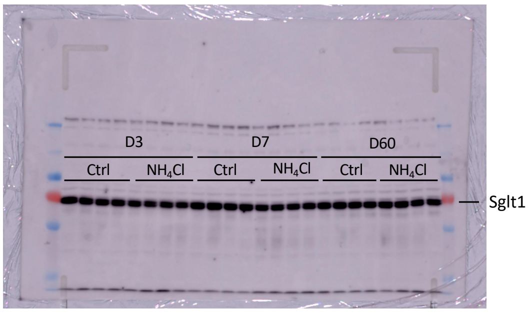
D3
D7
D60
Ctrl
NH₄Cl
Ctrl
NH₄Cl
Ctrl
NH₄Cl
Sglt1

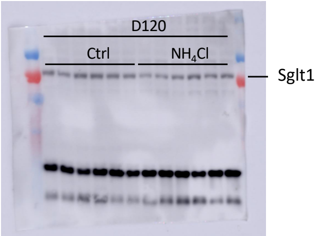
D120
Ctrl
NH₄Cl
Sglt1

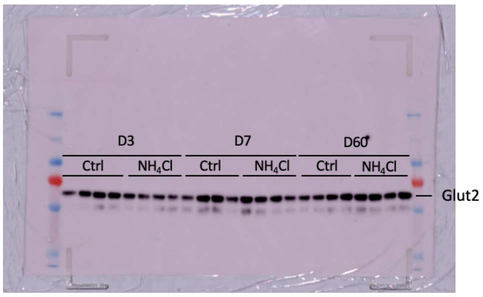
D3
D7
D60
Ctrl
NH₄Cl
Ctrl
NH₄Cl
Ctrl
NH₄Cl
Glut2

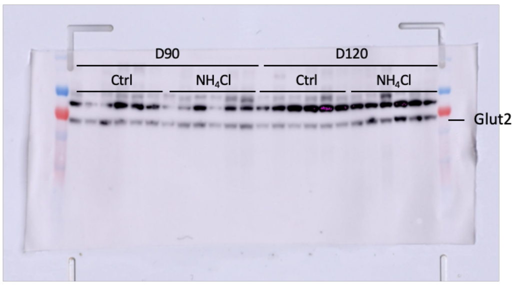
D90
D120
Ctrl
NH₄Cl
Ctrl
NH₄Cl
Glut2

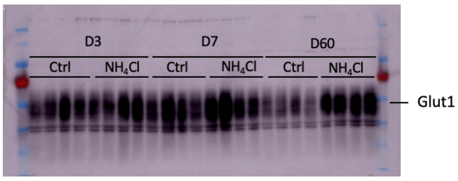
D3
D7
D60
Ctrl
NH₄Cl
Ctrl
NH₄Cl
Ctrl
NH₄Cl
Glut1

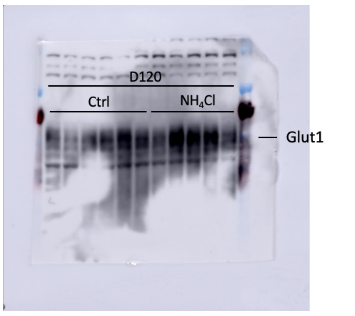
D120
Ctrl
NH₄Cl
Glut1

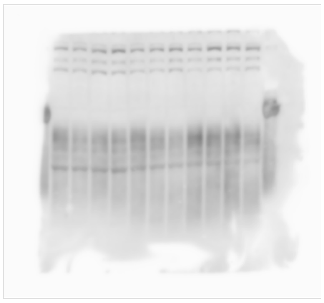

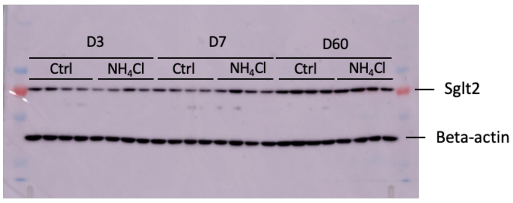

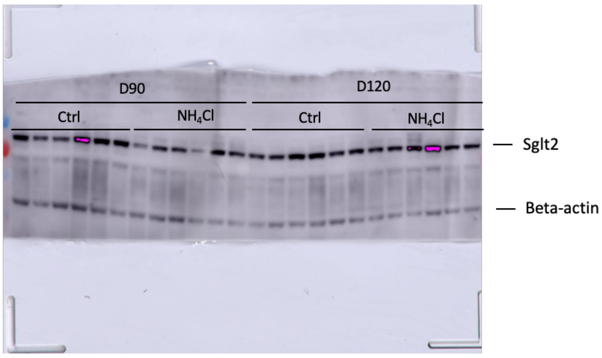

Supplementary Figure 13. **Full length western blots of Figure 5.G**
